# Relationship of Seat Interface Pressure to Change in Center of Pressure During Manual Wheelchair Pressure Redistribution Maneuvers

**DOI:** 10.3390/s25216507

**Published:** 2025-10-22

**Authors:** S. Andrea Sundaram, Andrew Hoang, Hannah Kuecker, Sivashankar Sivakanthan, Benjamin Gebrosky, Garrett G. Grindle, Cheng-Shiu Chung, Alicia Koontz, Brad E. Dicianno, Bradley S. Duerstock, Rosemarie Cooper, Rory A. Cooper

**Affiliations:** 1 Human Engineering Research Laboratories, School of Medicine, University of Pittsburgh, Pittsburgh, PA 15260, USA; akh55@pitt.edu (A.H.); hannahtkuecker@tamu.edu (H.K.); sis65@pitt.edu (S.S.); ggg3@pitt.edu (G.G.G.); joshua.chung.cs@pitt.edu (C.-S.C.); akoontz@pitt.edu (A.K.); dicianno@pitt.edu (B.E.D.); cooperrm@pitt.edu (R.C.); 2 Department of Rehabilitation Science and Technology, School of Health and Rehabilitation Sciences, University of Pittsburgh, Pittsburgh, PA 15260, USA; 3 U.S. Department of Veterans Affairs, Pittsburgh, PA 15260, USA; gebrosky@pitt.edu; 4 Department of Bioengineering, Swanson School of Engineering, University of Pittsburgh, Pittsburgh, PA 15260, USA; 5 Department of Physical Medicine and Rehabilitation, School of Medicine, University of Pittsburgh, Pittsburgh, PA 15260, USA; 6 Weldon School of Biomedical Engineering, Purdue University, West Lafayette, IN 47907, USA; duerstock@purdue.edu

**Keywords:** pressure injury, manual wheelchair, assistive technology

## Abstract

**Highlights:**

**What are the main findings?**

- As manual wheelchair users perform forward, leftward, and rightward leaning pressure redistribution maneuvers, seat interface pressure under the ischial tuberosi-ties decreases roughly linearly as the magnitude of the seated center of the pressure vector increases.
- The slope of the relationship between seat interface pressure and center of pressure magnitude varies by individual.

**What is the implication of the main finding?**

- Change in seated center of pressure can be used as a surrogate for measuring reduc-tion in seat interface pressure for wheelchair users executing pressure redistribution maneuvers.
- The most accurate results are obtained by determining the slope of the relationship for each individual.

**Abstract:**

Manual wheelchair users (MWUs) are at high risk of developing pressure injuries (PIs) from prolonged static sitting. Clinical practice guidelines suggest periodic pressure redistribution (PR) to mitigate this risk. Prior work has demonstrated that a wheelchair seat pan instrumented with force sensors can track the change in center of pressure (CoP) as MWUs perform PR and use this measurement to infer the direction and degree of a PR. This study’s objective was to quantify the relationship between change in CoP and reduction in seat interface pressure (SIP) under the ischial tuberosities for commonly practiced PR maneuvers. A theoretical model relating SIP and change in CoP for forward leaning PR was developed. Participants performed forward, leftward, and rightward leaning PRs while seated on a pressure mat on the test wheelchair with a load cell-instrumented seat pan. Linear mixed-effects models showed that the relationship of SIP and CoP varies by participant. Across participants, the change in SIP for a given change in CoP was greater with sideways than with forward leans. The type of cushion used did not affect the relationship. These findings can be used as part of her real-time smartphone-based coaching system for PI prevention.

## 1. Introduction

The World Health Organization (WHO) estimates that 1% of the world’s population (over 80 million individuals) requires a wheelchair [1]. In their lifetime, over half of wheelchair users will develop a pressure injury (PI), and the risk of PI is even higher for individuals with neurological conditions that impair movement and sensation (e.g., people with spinal cord injury (SCI), spina bifida, or multiple sclerosis) [2]. There are wide-ranging estimates on the prevalence of PIs in the SCI population. One study reported that each year, 25% of individuals with SCIs are likely to develop PIs [3], and a recent meta-analysis of 24 studies found a pooled prevalence of 32.36% [4]. PIs are often painful, harm overall health, and in instances of severe infection can result in death. To keep pressure off during healing, a wheelchair user may need to spend more time in bed—limiting work, social interaction, independence, and quality of life. PIs are also expensive to treat—costing $20,000–$150,000 per incident [5]. The etiology of PI formation is multifactorial—with temperature, moisture, nutrition, and many intrinsic factors being implicated [6,7]—but mechanical compression and shear of the skin, muscle, and fat tissues against bony prominences are widely accepted as the primary contributing factors that lead to cell death [8,9,10,11]. When seated, approximately 18% of body weight is concentrated over each ischial tuberosity (IT) and 5% over the sacrum [12]. Pressures in these regions often exceed the threshold of mechanical loading at which PI formation is thought to begin, causing capillary occlusion, cell deformation, and, over time, tissue damage [13].

Proper wheelchair setup and weight redistributing cushions can help reduce the incidence of PIs [13], but no cushion can completely eliminate sitting pressure. Although there appears to be a qualitative relationship between measured seat interface pressure (SIP) and PI formation, there is no clinically relevant threshold that predicts PI development [14]. This may be because SIPs significantly underestimate internal pressures at the bony prominences [15,16,17]. Leaning the torso in the frontal and sagittal planes have been shown to reduce pressures under the ITs [18,19], and necessarily change the direction of forces at the bone–muscle interface, changing the tissues that are exposed to high pressures.

For this reason, clinical practice guidelines (CPGs) recommend periodic pressure redistribution (PR) in addition to pressure-distributing seating to manage sitting pressures [7,20]. Individuals who have sufficient strength and trunk stability are advised to perform PRs such as forward and lateral leaning as well as wheelies every 15 to 30 min in order to reduce prolonged exposure of any area to high mechanical loading [7,20,21]. Training in these PR techniques is typically provided by clinicians through oral instruction, sometimes with the help of a pressure mapping system [22,23], but there is little opportunity to reinforce these lessons with wheelchair users outside of the clinic, and studies have shown that MWUs rarely adhere to this advice [20,24,25,26]. Wheelchair users have expressed interest in being coached in PI prevention [27,28] including an openness to using apps and connected instrumentation [29,30]. A system for coaching power wheelchair users proved effective in increasing the appropriate use of their power seating functions [27,31]. The system used accelerometers to determine the relative angles of the wheelchair seat’s components, but tracking the seated position of MWUs is more challenging. Over the past several decades, systems to track MWUs’ PR maneuvers have been developed using mechanical switches, pressure sensitive mats, and other sensors [32]. However, pressure mats are too fragile for use in community environments and any system involving pressure sensors will have differing responses with different cushion interfaces [33].

An alternative approach uses load cells under the wheelchair’s seat pan. Load cells are more robust to environmental factors than are pressure mats, and because they are measuring forces rather than pressures, they should not vary in their response with different cushions. A system that used a seat pan instrumented with load cells to measure MWUs’ change in seated center of pressure (CoP) and used CoP to classify forward, leftward, and rightward leaning PRs has been previously described [30]. Briefly, if the system measured the user’s CoP near the center of the seat pan, it inferred that the user was in a neutral sitting posture. If the CoP moved forward or to either side beyond a threshold, the user was thought to be attempting a PR. Separately, a reduction in total weight on the seat was used to infer a push-up. These thresholds were determined by observing the changes in CoP when MWUs were asked to attempt these PR maneuvers but were not compared to any other measure of PR effectiveness [34].

The obvious criterion for when an MWU is performing an effective PR is a substantial reduction in pressure under one or both ITs). Short of 100% offloading, however, there is no accepted standard for what proportion of offloading constitutes effectiveness. One study found that even a 30% reduction in pressure improved blood flow [35], but clinicians generally encourage MWUs to reduce pressure as much as possible when executing a PR.

Our objective was to relate the change in CoP measured by our system to the proportional reduction in pressure at the ITs. We first present a theoretical model that predicts how the CoP changes as an individual executes forward and sideways leans. We then derive an equation that relates the change in proportional pressure under the ITs to changes in the user’s CoP. We verify our theoretical relationship by simulating a model of a wheelchair test dummy executing a PR. Finally, the theoretical framework is used as the basis for developing a model of the relationship from human subject test data.

Although pressures would ideally be measured at the bone–muscle interface, where deep pressure injuries are thought to begin [8], it is difficult to measure compression in these areas without using magnetic resonance imaging [36], which would be incompatible with the load cell-based measurement system. Researchers and clinicians most often use pressure mats to measure SIP, the results of which can be extrapolated to the bone–muscle interface [15]. We follow this approach so that the final model relates changes in proportional SIP to changes in CoP.

### 1.1. The Mathematical Model

**Figure 1:**
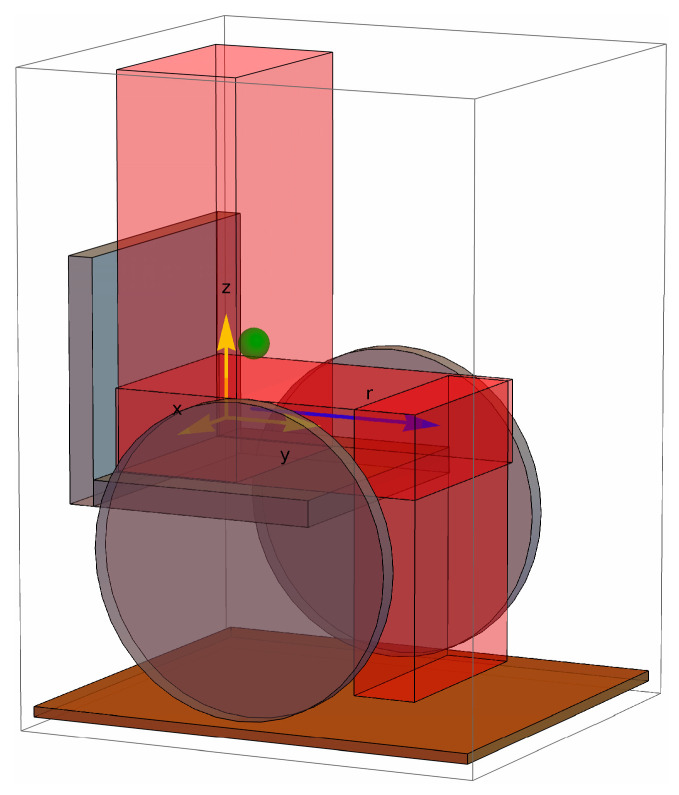
Representation of an upright-seated MWU with markings for the coordinate system and axis of leftward leans (r).

The CoP of the user on the wheelchair’s seat is the projection of the user’s center of mass (CoM) along the gravity vector where it intersects the seating surface. If the wheelchair is on level ground, the CoP is at the point on the wheelchair’s seat that is directly below the CoM. For the purposes of relating seated posture with CoP, the most basic model can represent the wheelchair user as consisting of three segments—trunk, upper legs, and lower legs—connected by hinged joints. The user’s CoM is simply the superposition of the CoMs of these three segments (Figure 1).

In the following equations, the x, y, and z dimensions are defined, respectively, as left–right, back–front, and bottom–top, relative to the wheelchair user, with the origin located at the left–right midpoint along the hinged joint joining the trunk and upper legs, near the sacrum.

### 1.2. Modeling Seated Center of Pressure

The locations of the centers of mass of the three body segments can be given as vectors $\vec{T}$, $\vec{U}$, and $\vec{L}$, respectively, representing the CoM of the trunk, upper legs, and lower legs when the wheelchair user is seated upright. However, in a properly set up wheelchair, the lower legs will be supported by the footrests and, therefore, not contribute to the mass supported by the seat. If the proportion of body mass of the trunk and upper legs are t_m_ and u_m_, then the relevant CoM for the upright-seated user is simply the vector $\vec{T}\;\cdot \;$t_m_ $+\;\vec{U}\cdot \;$u_m_.

In a forward lean, the trunk segment will be rotated about the x-axis in the negative direction through an angle *θ* In a leftward lean, both trunk and upper leg segments will be rotated about the left femur in the negative direction through an angle ϕ. The left femur can be thought of as extending in the y-direction from a point on the x-axis a distance, r, to the left of the body coordinate system’s origin. The rotation of a leftward lean is then calculated as occurring about a vector parallel to the y-axis with its origin at the point (−r, 0, 0). The CoM is therefore given by Equation (1).

(1) $$\left(\begin{array}{cccc} Cos\left[\phi \right] & Sin\left[\theta \right]Sin\left[\phi \right] & Cos\left[\theta \right]Sin\left[\phi \right] & -r+rCos\left[\phi \right]\\ 0 & Cos\left[\theta \right] & -Sin\left[\theta \right] & 0\\ -Sin\left[\phi \right] & Cos\left[\phi \right]Sin\left[\theta \right] & Cos\left[\theta \right]Cos\left[\phi \right] & -rSin\left[\phi \right]\\ 0 & 0 & 0 & 1 \end{array}\right)\left(\vec{T}\;\cdot t_{m}\right)+\left(\begin{array}{cccc} Cos\left[\phi \right] & 0 & Sin\left[\phi \right] & -r+rCos\left[\phi \right]\\ 0 & 1 & 0 & 0\\ -Sin\left[\phi \right] & 0 & Cos\left[\phi \right] & -rSin\left[\phi \right]\\ 0 & 0 & 0 & 1 \end{array}\right)\left(\vec{U}\cdot \;u_{m}\right)$$

Equation (1): Body center of mass for forward and leftward lean.

**Figure 2:**
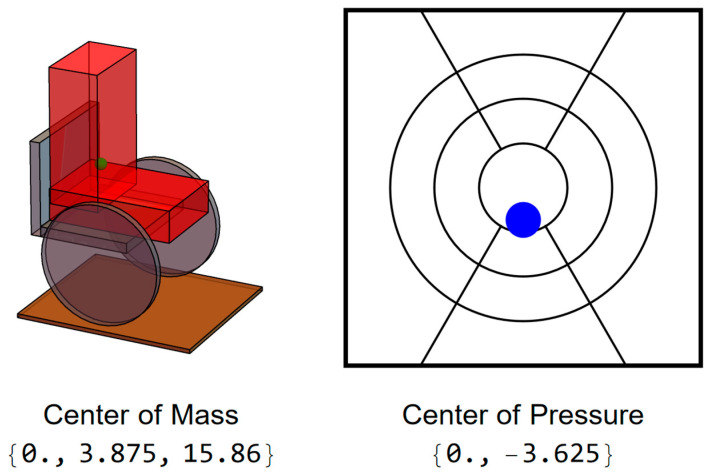
Representation of an upright-seated user with corresponding display of CoP. The green sphere on the left represents the user’s center of mass, and the blue dot on the right represents the user’s center of pressure.

For the following illustrations, the measurements of each segment were taken from a 165 cm tall female, the CoM of each body segment was assumed to be at its geometric center, and the weight distribution between the segments used the instructions for dummy construction in the protocols for wheelchair testing, ISO 7176-11 [37]. If the wheelchair is on level ground, the x-coordinate of the CoP will be equal to the x-coordinate of the CoM while the y-coordinate will be displaced by the distance between the body coordinate system and wheelchair seat coordinate system, equal to half the seat depth minus half the trunk depth. When the user is in the upright (neutral) position, the CoP will be at midline from left to right and slightly behind the center of the wheelchair’s seat (Figure 2).

**Figure 3:**
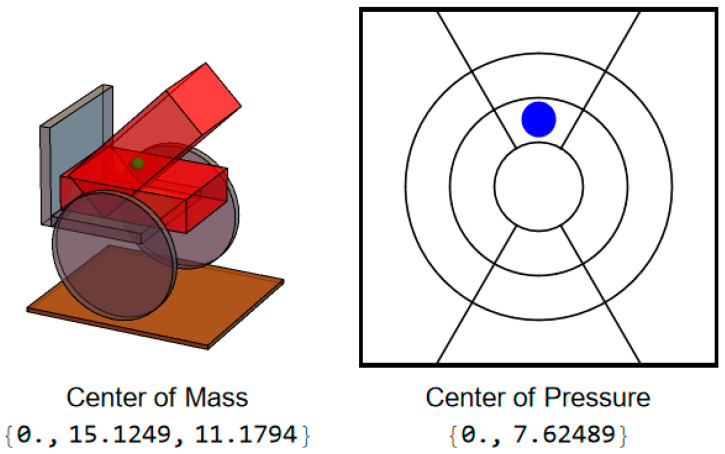
User position and CoP during forward lean.

When executing a forward lean, the user’s CoM will remain at midline but move in the positive y-direction (Figure 3).

**Figure 4:**
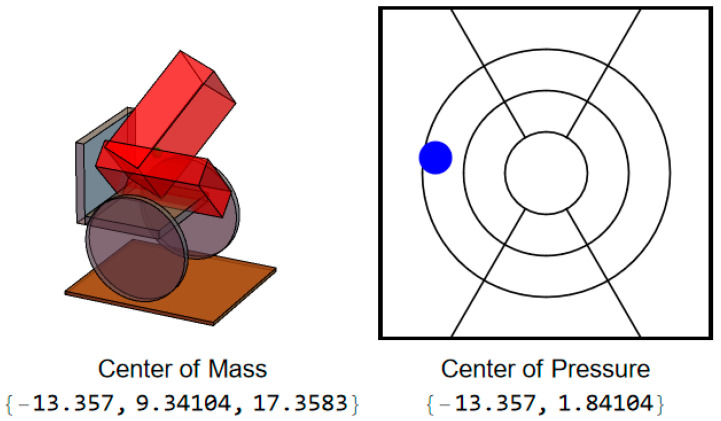
User position and CoP during leftward lean.

During a leftward leaning PR, the CoMs of the trunk and legs will both move to the left, but this PR also typically incorporates some degree of forward lean of the trunk, which will result in the user’s CoM, and thus CoP, moving not only to the left but forward as well (Figure 4).

The extent of a lean in any direction can be represented by the magnitude of the vector of the change in CoP, which, in the simplest case, is that of a forward lean; if we assume $\vec{T}$ is initially purely in the Z direction, the CoP magnitude is given by Equation (2):

(2) $$\left(\left|\vec{T}|\cdot t_{m}\cdot sin(\theta \right)\right)$$

Equation (2): Center of pressure magnitude for a forward lean.

For a leftward-leaning PR, if we likewise assume $\vec{U}$ is initially purely in the ydirection, the CoP magnitude is given by Equation (3):

(3) $$\sqrt{{\left|\vec{T}\right|}^{2}{t_{m}}^{2}{sin}^{2}\left(\theta \right)+{(-r\left(t_{m}+u_{m}\right)+r\left(t_{m}+u_{m}\right)\cos\left(\phi \right)+|\vec{T}|t_{m}\cos\left(\theta \right)\sin\left(\phi \right))}^{2}}$$

Equation (3): Center of pressure magnitude for a combined forward and leftward lean.

### 1.3. Determining Pressure Under the Ischial Tuberosities

To calculate the pressure on the ITs during a forward lean, we can consider the torso and upper leg segments to be supported on a surface with uniform elasticity. We continue to consider the torso and upper leg segments as rigid blocks connected by a hinged joint and allow the distal end of the upper leg segment (at the knees) to move, accounting for it sinking into the cushion as the CoM moves in its direction. The restoring force of the surface must satisfy the equilibrium equations for force and torque at every point along y. If f(y) is the force at any point along y, the upper leg segment has length L, the total mass of the torso and upper leg segments is m, and CoM_y_ is the user’s center of mass in the y-dimension; the force and moment equations for static equilibrium are, respectively, Equations (4) and (5).

(4) $$\int_{0}^{L}f\left(y\right)\cdot dy=m\cdot g$$

Equation (4): Force static equilibrium.

(5) $$\int_{0}^{L}f\left(y\right)\cdot y\cdot dy={COM}_{y}\cdot m\cdot g$$

Equation (5): Torque static equilibrium.

If the upper leg segment is considered as a non-deforming beam, f(y) must be continuous and can be substituted with ay + b, giving Equations (6) and (7).

(6) $$\int_{0}^{L}\left(ay+b\right)dy=m\cdot g$$

Equation (6): Linear distribution of restoring force.

(7) $$\int_{0}^{L}\left(ay+b\right)y\cdot dy={CoM}_{y}\cdot m\cdot g$$

Equation (7): Linear distribution of restoring torque.

Solving for a and b and substituting back into the equation for f(y) yields Equation (8).

(8) $$f\left(y\right)=-2gm(\frac{\left(3{CoM}_{y}-2L\right)}{L^{2}}-\frac{3\left({2CoM}_{y}-L\right)y}{L^{3}})$$

Equation (8): Restoring force at any point along y for static equilibrium.

The proportional pressure is the pressure under the IT(s) at any point during a lean divided by the pressure when the user is sitting upright. Equation (8) can be used to calculate the proportional pressure as CoM_y_ changes. If CoM_y0_ is the CoM in the y-dimension when the user is sitting upright, the proportional pressure as a function of the change in CoM_y_ is Equation (9):

(9) $$p\left(∆{CoM}_{y}\right)=1+\left(\frac{3(L-2y)}{{3CoM}_{y0}\left(L-2y\right)-L(2L-3y)}\right)∆{CoM}_{y}$$

Equation (9): Proportional pressure at y as a function of change in CoM_y_.

The position where the pressure is being evaluated, under the ITs, is represented by y. Examining Equation (9), the slope of the proportional change in SIP with increasing CoP will be steeper as the initial CoM_y_ when sitting upright moves further away from the trunk, the measurement position moves closer to the hip joint, or the upper leg segment is shorter.

If the pressure is evaluated directly under where the torso joins the upper legs, y = 0, then the proportional SIP simplifies to Equation (10).

(10) $$p\left(∆{CoM}_{y}\right)=1+\left(\frac{3}{{3CoM}_{y0}-2L}\right)∆{CoM}_{y}$$

Equation (10): Proportional pressure at y = 0 as a function of change in CoM_y_.

In the case of a lean purely in the forward direction, $∆{CoM}_{y}$ is equal to the magnitude of the vector representing change in CoP.

Following the simple model presented, a leftward lean would lift the right IT immediately off the seat or at least contact between the IT and the seat would cease when the right IT had been lifted beyond the combined initial compression of the seat material and tissues. Alternatively, depending on the movement of the user’s spine, the CoP may shift to the left without lifting of the right side, which could be modeled similarly to the forward lean. Either approach requires assumptions beyond what can be verified in the current study, but both approaches will produce a larger change in proportional SIP for a given change in CoP than occurs during a forward lean.

### 1.4. STATIC Force Simulation

**Figure 5:**
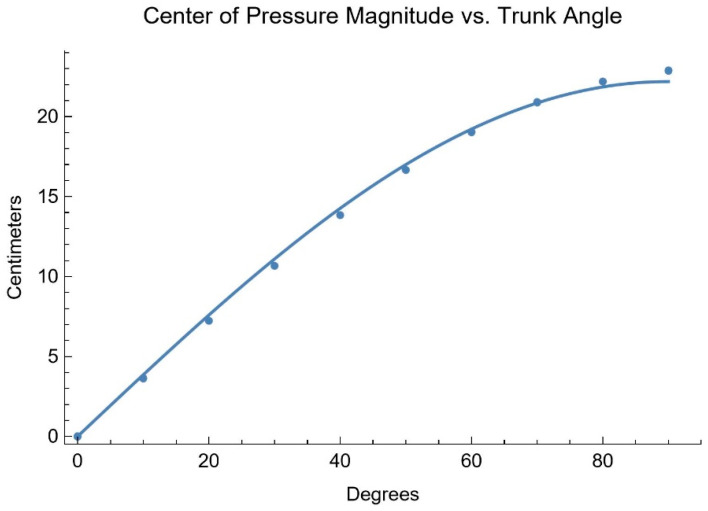
Simulation results of CoP magnitude at different trunk angles.

To examine the expected shapes of the response curves, a static force analysis was performed in SolidWorks 2021 (SolidWorks Corp., Waltham, MA, USA) by positioning a 3D model of the test dummy specified in ISO 7176-11 atop a horizontal surface representing the wheelchair’s seat. The simulation measured the downward force in an area equivalent to the locations of the ITs while the angle between the upper body and upper leg segments of the model decreased in intervals of 10°. The three-dimensional CoM was also recorded at each angle interval (Figure 5). For this purely forward lean, the magnitude of the CoP vector is simply the difference in CoM in the direction of lean.

Based on the mathematical model, CoP magnitude versus trunk angle data should fit Equation (11).

(11) $${CoP}_{mag}=b\cdot sin(\theta )$$

Equation (11): Center of pressure magnitude for a forward lean.

Fitting the data to Equation (11) gives ${CoP}_{mag}=22.196\cdot sin(\theta )$ with an R^2^ of 0.9995.

**Figure 6:**
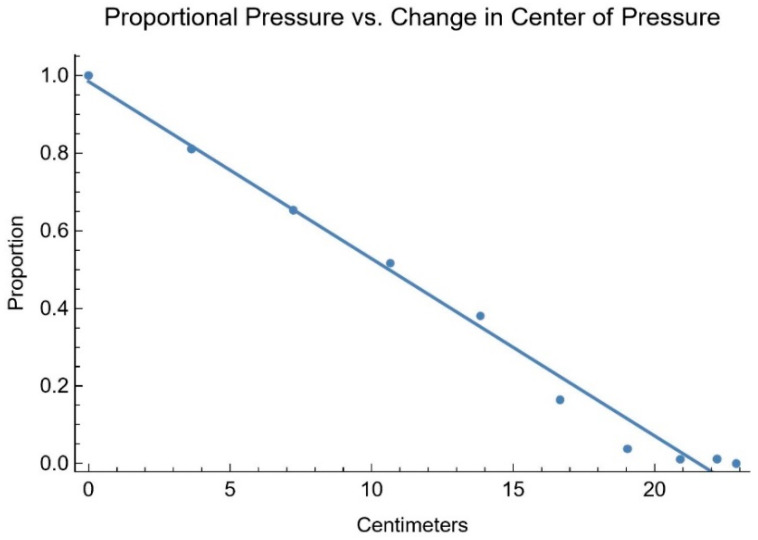
Simulation results of proportional IT pressure verses CoP magnitude.

Fitting the simulation results (Figure 6) to a first-order linear equation, consistent with the mathematical model, gives proportion 0.9844 − 0.0457 $\cdot \;{CoP}_{mag}$ with an R^2^ of 0.9866.

The good fit of these equations with the static force simulation data supports the usage of equations of these forms when fitting the experimental user data.

## 2. Methods

### 2.1. Experimental Setup

The apparatus for measuring CoP comprised a Permobil TiLite ZRA Ultralight manual wheelchair with a 40 cm × 40 cm, 6.35 mm thick aluminum seat pan supported at its 4 corners by 50 kg Phidget 3135 single-point load cells (Phidget Inc., Calgary, AB, Canada) mounted to the wheelchair’s frame. The wheelchair was set up with a seat dump angle of 8° and a backrest angle of 10°. Analog voltage measurements from the load cells were digitized at a sample rate of 125 Hz by a 4-channel Phidget Bridge 1046 USB interface with data collected by a custom Python version 3.11 script. The load cells were calibrated in situ by stacking weights at the center of the seat pan while the wheelchair’s frame was held level. The CoP was calculated according to Equation (12).

(12) $${CoP}_{x}=\frac{w\left(\left(F_{FR}+F_{BR}\right)-\left(F_{FL}+F_{BL}\right)\right)}{2(\left(F_{FR}+F_{BR}\right)+\left(F_{FL}+F_{BL}\right))},{CoP}_{y}=\frac{d(\left(F_{FL}+F_{FR}\right)-\left(F_{BL}+F_{BR}\right))}{2(\left(F_{FR}+F_{BR}\right)+\left(F_{FL}+F_{BL}\right))}$$

Equation (12): Center of pressure for -–cornerplaced load cells.

*F_BL_*, *F_BR_*, *F_FL_*, and *F_FR_*, represent the forces at the back-left, back-right, front-left, and front-right of the seat pan, respectively, and *d* and *w* represent the depth and width of the seat pan measured between the load cells. With the wheelchair on level ground, the seat pan made an angle of 8° with the horizontal plane. In this orientation, by stacking weights at known locations on the seat pan, the maximum error in CoP was found to be 1.96 cm with all weight concentrated at the extreme back edge of the seat pan. Error in CoP toward the center of the seat pan where participants’ CoPs are likely to be located was less than 1 cm.

An XSENS motion capture system (Movella Inc., Henderson, NV, USA) was used to track participant lean angles. Inertial measurement units (IMUs) were strapped or adhered with tape to each participant’s sternum, pelvis (on the posterior iliac spine), and the midpoint of each femur. A fifth IMU was fixed to the back-right corner of the wheelchair cushion. The XSENS system acquires data at a sample rate of 100 Hz. Data was collected using the system’s dedicated MT Manager version 2022.0 software. To synchronize XSENS and load cell data acquisition, a TTL trigger from the XSENS base station-initiated load cell data collection on the Python script was used.

A BodiTrak2 LT pressure mapping system (Vista Medical Ltd., Winnipeg, MB, Canada) with a 16 × 16 array of pressure-sensing elements, each measuring 6.45 cm^2^, recorded SIP. Using its proprietary BodiTrak Pro 6.0 software, the pressure mat acquires data at a nominal sample rate of 15 Hz, although loads from computer background tasks slowed data acquisition on some trials. The pressure mat ran continuously throughout all of the PR maneuvers for each participant, and system timestamps were used during postprocessing to align the pressure mat and load cell data.

For measurement of body mass distribution, a BodiTrak 2 Pro Bed Sensing Mat with an array of 27 × 64 sensing elements, each measuring 6.45 cm^2^, was placed atop either an air mattress or a deep immersion phone mattress. Using its proprietary software, a single frame of data was saved for subsequent analysis. Factory calibrations were used for both pressure mats.

### 2.2. Study Procedure

This study was approved by the University of Pittsburgh Institutional Review Board (IRB #23010214). A convenience sample of 10 able-bodied participants and 10 participants who use manual wheelchairs was used. Inclusion criteria for the MWUs were to be age 18 or older, have a condition affecting the spinal cord, use a manual wheelchair at least 30 h/week, weigh less than 113 kg (design limit of the testing device), have no active gluteal or thigh pressure injuries, and be able to perform forward, leftward, and rightward PR maneuvers. Inclusion criteria for the able-bodied participants were to be age 18 or older, not have a condition affecting the spinal cord, not use a wheelchair, weigh less than 113 kg, and be able to perform the PR maneuvers. After informed consent was obtained, participants were weighed, then asked to transfer onto the bed pressure mat. After allowing 5 min for the participant to become immersed, a frame of the pressure mat values was saved. During that time, lengths of participants’ lower legs, upper legs, lower torso, upper torso, and head segments were measured (landmarks at calcaneus, mid patella, anterior superior iliac spine, xyphoid process, sternal clavicular joint, and vertex) to segment sections of the body for determining mass distribution.

The cushion to be tested was then placed on the test wheelchair and the wheelchair pressure mat was positioned atop it. Able-bodied participants completed the study with both a Varilite Evolution air cushion and a J Basic Wheelchair Cushion foam cushion, whereas MWUs were invited to use their own cushions. Pressure mat recording was started, and the Python script was triggered to acquire data for later zeroing. Participants were then asked to transfer to the study wheelchair and the IMUs were attached. If the participant’s feet did not reach the footrests, blocks were added to approximate the appropriate set up. They were then asked to sit still for 30 s while data was acquired. A clinician instructed participants on proper PR technique. Subsequently, they were asked to perform forward, leftward, and rightward leans as slowly as possible and to the maximum extent they were comfortable doing so. Each PR maneuver was recorded separately with the participant being told when they could begin and informing the study personnel when they reached their maximum lean. The sequence of forward, leftward, and rightward leans was then repeated 2 additional times. Study personnel were positioned to assist the participants in case they were to lose their balance. Able-bodied participants repeated the protocol twice with each cushion: once from sitting upright and once starting at the backrest. MWUs all started at the backrest as few had the trunk stability to independently sit upright.

### 2.3. Data Analysis

To determine the mass distribution of each participant, the previously saved pressure map from the bed-sized mat was loaded into the BodiTrak software. The body segment measurements taken while the participant was supine were mapped onto the sensor grid—the center-to-center distance between grid elements measured 2.86 cm in both directions. The BodiTrak software can report a value in Newtons for a selected region of interest based on summing the pressures over the included cells; these were used to calculate the mass of each body segment. Additionally, the reported center of pressure of the segments was used to determine the center of mass location of the upper body segment and upper legs segment relative to the ASIS.

Most other data analysis was performed in MATLAB R 2024b (The MathWorks, Natick, MA, USA). Analysis of linear models was performed in R Studio 2025 (Posit, Boston, MA, USA). Bland–Altman plots were generated in Microsoft Excel (Microsoft Corporation, Redmond, WA, USA). For each combination of participant–cushion–starting position, the wheelchair pressure mat data was imported, and the timestamps from the load cell files were used to segment each test. To define the area under each IT, a heat map was generated from the static sitting test and the indices of the 4 contiguous cells of highest pressure in the area of each IT were recorded. In those cases where more than 4 cells had high pressures, the first forward-leaning PR test was used to identify the areas where the pressures stayed high the longest. In the subsequent analysis, the pressure measurements of the 4 cells were averaged and that value was taken as the pressure under the corresponding IT. Load cell measurements taken at the beginning of each test series before the participant had transferred to the wheelchair were used to subtract the weight of the cushion plus pressure mat so that the load cells only measured participant weight distribution.

The start time from each test’s load cell file and IMU sample rate were used to generate timestamps for the IMU data. For initial data analysis, the means of the first half-second of data for each test were used to calculate the zeros from which angle changes were measured, the starting point of the CoP vector, and the mean pressures under each IT against which proportional pressures were measured. As the pressure mat had the lowest sample rate, its timestamps were used to find the closest measurements from the load cells and IMUs for each pressure mat measurement. The maximum time difference between corresponding samples from the different measurement systems was less than 5 ms. The synchronized data was then used to graph the results for each participant for each test.

To normalize the proportional pressure response across starting conditions, the maximum of the mean pressure under both ITs was found for each test. All pressures for that test were divided by the maximum mean pressure. The maximum mean pressure should occur when the participant is sitting upright. Changes in CoP and angles were then recalculated from the time of maximum mean pressure; this did not alter the data for the tests that began in the upright-seated position.

For each experimental condition—e.g., foam cushion–backrest starting position–forward lean—a regression line was fitted to the combined data from the 3 repeated trials for each participant, which provided the relationship between CoP magnitude and proportional pressure. To exclude the extreme ends of the leans where the response becomes nonlinear and reaches a plateau on some trials, only data from the first 95% of the SIP reduction was included. For the AB participants, regression lines were also fitted for the combined forward lean data, i.e., both cushion types and both starting positions. Participant body segment measurements were used in the equation for the mathematical model to calculate the expected slopes for forward leans for comparison. Bland–Altman plots were constructed comparing the difference between fitted and theoretical slopes to determine whether they varied in any systematic way.

The same generally linear subset of the data was used to construct multiple linear mixed-effects models. In all models, proportional pressure was the response variable, CoP magnitude was a continuous fixed effect, PR direction (forward, left, or right) was a categorical fixed effect, and participant was a categorical random effect. Models were centered on the mean CoP. For the AB participants, cushion type (air or foam) and starting position (sitting upper backrest) were additional categorical fixed effects. Initially, separate models were constructed for the AB and MWU participants. Linear mixed-effects models are robust to violations of normality of residuals and homoscedasticity, provided the underlying structure of the data is appropriate to the analysis [38,39]. In order to account for the hierarchical structure of the data, Satterthwaite approximation was used to calculate degrees of freedom [40]. Several different random effects structures were evaluated, and the Akaike information criterion (AIC) and Bayesian information criterion (BIC) were used to determine which best represented the underlying processes [41]. Analyses focused on CoP and its interaction with other factors because without a change in CoP, there can be no change in SIP.

Subsequently, additional models were evaluated that included both AB and MWU participants. As all MWU participants began their leans from the backrest, only AB participant data from backrest starting position was used. In addition to testing different random effects structures, models were evaluated that did and did not include participant type as a categorical fixed effect to determine whether there was a systematic difference between the AB and MWU participants. Again, AIC and BIC were used to determine which model best represented the underlying processes.

To examine the relationship between trunk lean angle and CoP magnitude, the sternum pitch was used to fit the equation CoP = b ∙ sin (sternum pitch), as derived in the mathematical model, for each experimental condition of the forward leans—e.g., foam cushion and backrest starting position. The XSENS sensors have a maximum range of 180°. To avoid crossing the 0° boundary at the beginning of the lean, the sensors were oriented such that upright-sitting registered approximately 90°. In the case of extreme forward leans, it was therefore possible to go beyond the 180° position and introduce a discontinuity in the data. To prevent these discontinuities or the asymptotic behavior at the extremity of some forward leans from distorting the fitted curve, only the first 90% of each trial was used to fit the equation. The theoretical value for b, based on the mathematical model, was calculated for each participant from the body segment lengths and mass distribution measurements. Bland–Altman plots were constructed comparing the differences between the fitted and theoretical coefficients.

## 3. Results

Able-bodied participants were four males and six females, with a mean age of 20.5 years (range: 20–22 years), a mean height of 171.7 cm (range: 157.5–185.4 cm), and a mean weight of 73.5 kg (range: 51.3–110.9 kg). MWUs were seven males and three females, with a mean age of 39.1 years (range: 29–56 years), a mean height of 168.5 cm (range: 160.0–182.9 cm), and a mean weight of 79.3 kg (range: 45.8–119.1 kg), who had been using a wheelchair for an average of 22.85 years (range: 1.5–42 years). (Note that the weight of one participant slightly exceeded the eligibility criteria because eligibility screening was based on self-reported weight. However, the experimental setup had been tested for safety beyond 140 kg.) They reported their diagnoses as SCI T11, spina bifida, SCI complete T10–11, T10 complete paraplegia, paralysis SCI, SCI: T11–T12, paraplegic, amniotic band syndrome, post laminectomy with neuropathy, and SCI.

### 3.1. Single Subject

**Figure 7:**
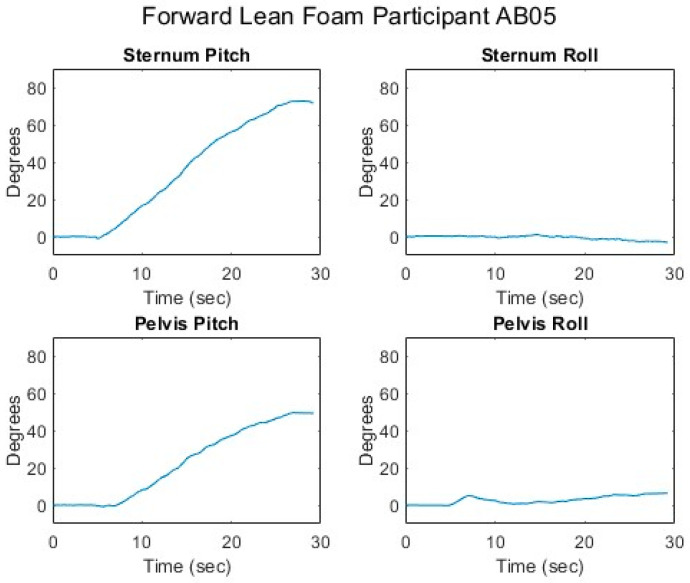
Upper body angles while executing a forward lean for an AB participant.

**Figure 8:**
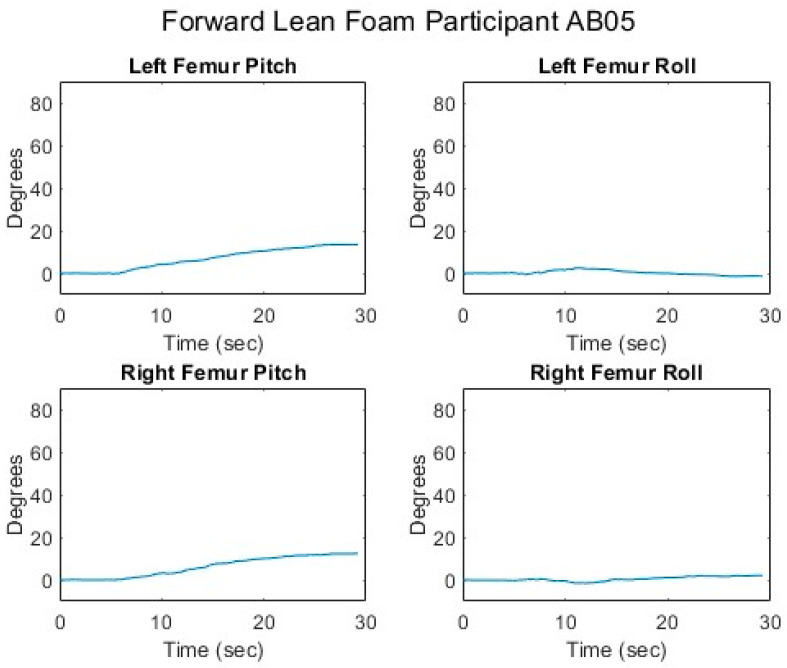
Femur angles while executing a forward lean for an AB participant.

**Figure 9:**
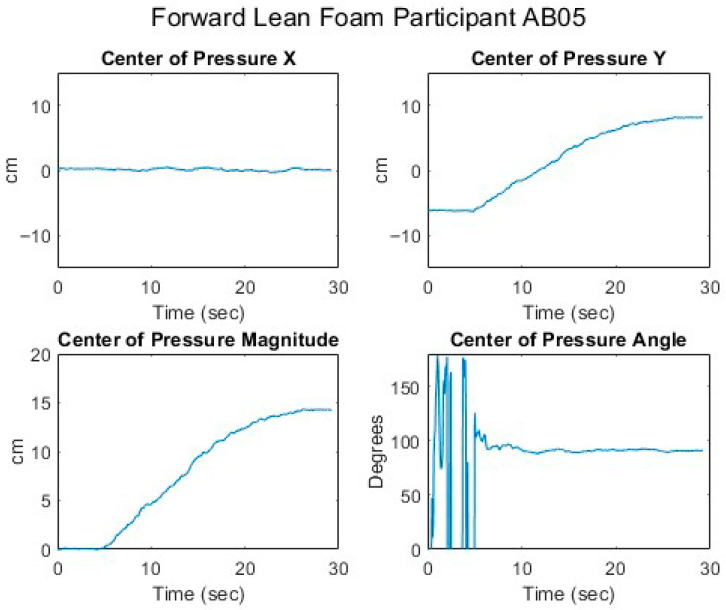
Seated center of pressure while executing a forward lean for an AB participant.

**Figure 10:**
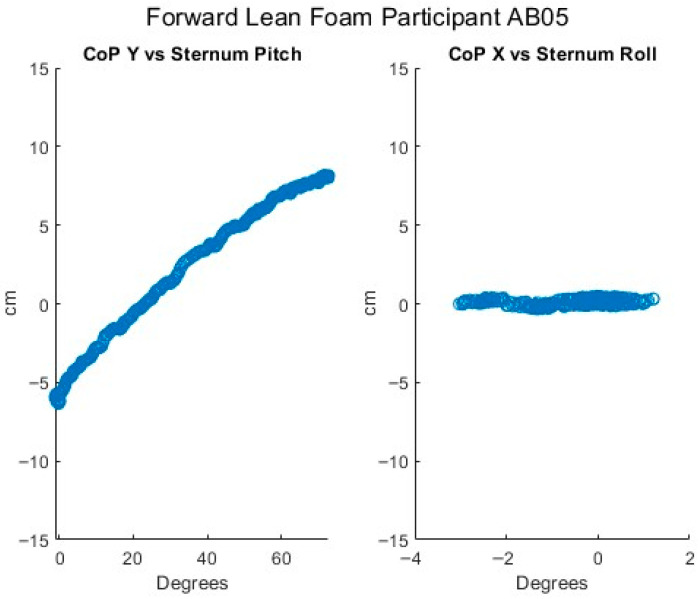
Relationship of center of pressure to pitch and roll angles while executing a forward lean for an AB participant.

**Figure 11:**
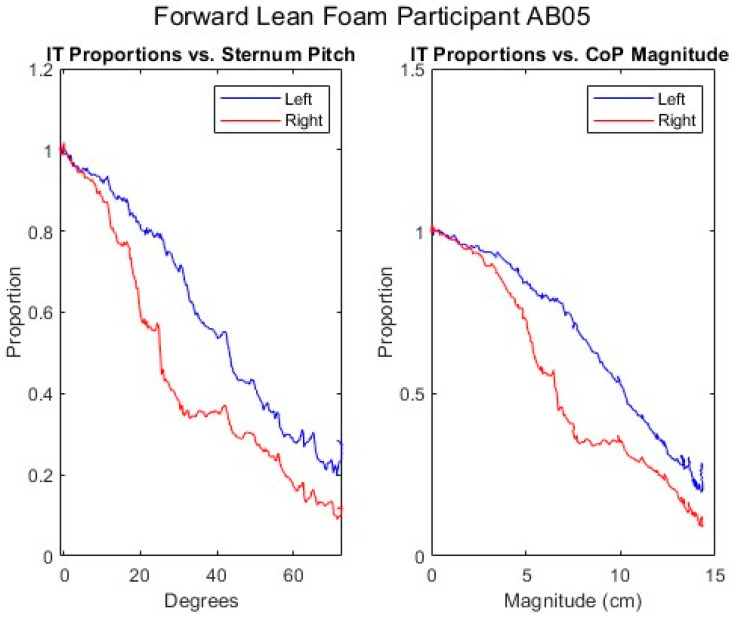
Proportional IT pressures while executing a forward lean for an AB participant.

Figure 7, Figure 8, Figure 9, Figure 10 and Figure 11 present data from one of the able-bodied participants performing a forward-leaning PR beginning from upright-sitting while using the foam cushion. These results are broadly representative of what was observed for the able-bodied participants.

Figure 7 shows that when executing a forward lean, both sternum and pelvis pitch generally increase while there is little change in their respective roll angles. The increase in sternum pitch is greater than the increase in pelvis pitch, suggesting some curvature of the spine. Figure 8 shows that the pitch of both femurs increases somewhat while the role is essentially unchanged. Figure 9 shows that the CoP in the y-direction (back–front) begins behind the center of the seat pan and moves smoothly forward while the CoP in the x direction (left–right) has little change. The magnitude of the CoP vector is therefore approximately the same as the change in CoP_y_ and its angle is close to 90°. Note that when the CoP vectors magnitude is close to zero, small shifts in the direction cause significant changes in the angle. Figure 10 shows that CoP_y_ increases as the sternum pitch increases while the negligible change in CoP_x_ reflects the negligible change in sternum roll. Figure 11 shows that the proportional pressures under both ITs decrease as the compound sternum angle and CoP magnitude increase.

**Figure 12:**
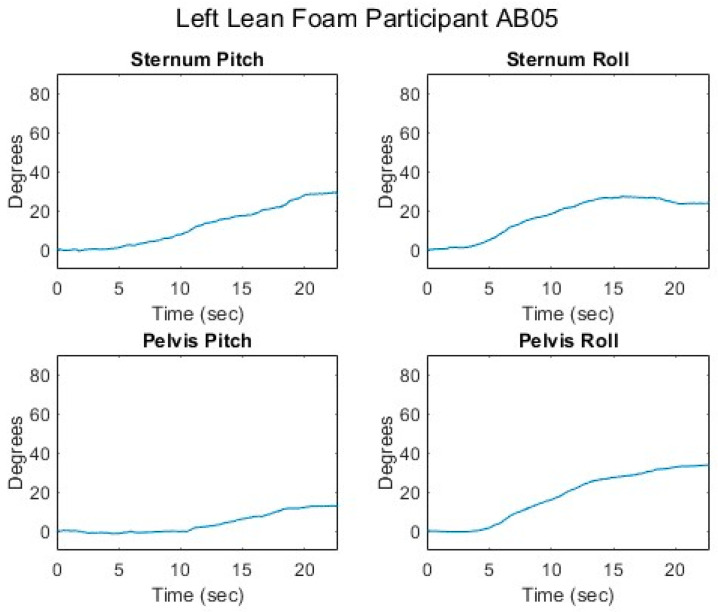
Upper body angles while executing a left lean for an AB participant.

**Figure 13:**
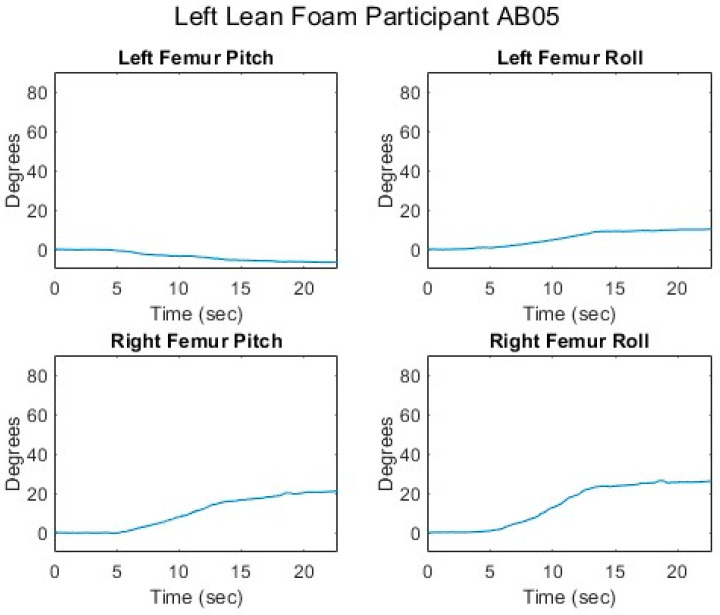
Femur angles while executing a left lean for an AB participant.

**Figure 14:**
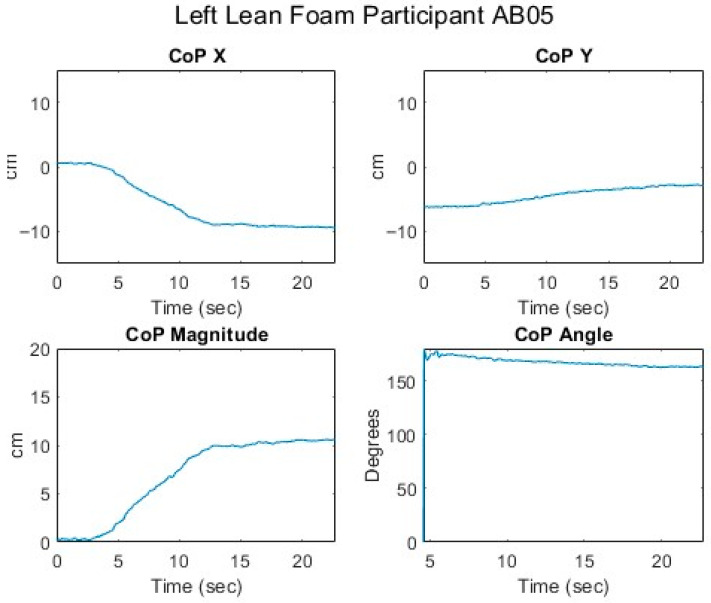
Seated center of pressure while executing a left lean for an AB participant.

**Figure 15:**
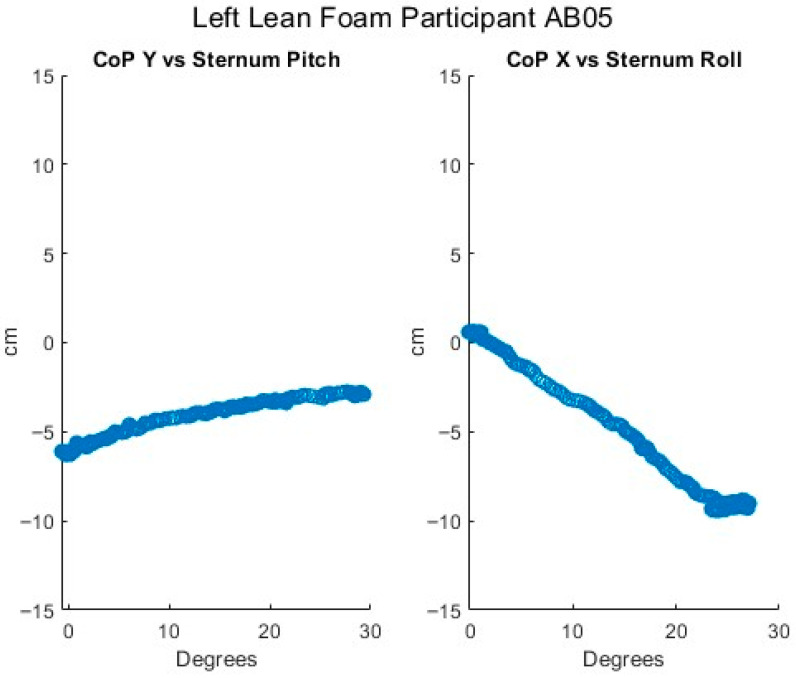
Relationship of center of pressure to pitch and roll angles while executing a left lean for an AB participant.

**Figure 16:**
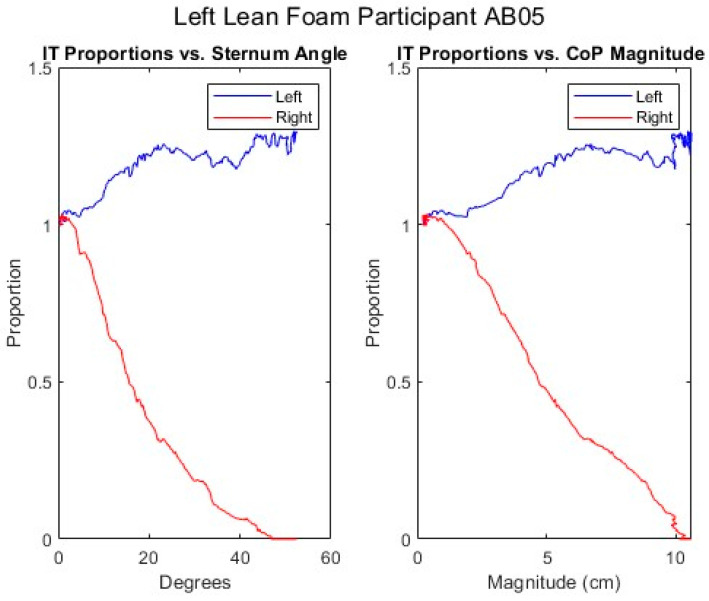
Proportional IT pressures while executing a left lean for an AB participant.

Figure 12, Figure 13, Figure 14, Figure 15 and Figure 16 present data from the same able-bodied participant performing a leftward leaning PR beginning from upright-sitting while using the foam cushion. These results are broadly representative of what was observed for the able-bodied participants.

Figure 12 shows that while executing a left lean, the upper body changes in both pitch and roll, reflecting the participant leaning forward while they are leaning to the left. The greater change in sternum pitch compared to pelvis pitch again suggests some curvature of the spine with the top of the upper body leaning forward more than the lower portion of the upper body. The greater change in pelvis roll compared to sternum roll likely reflects the participant curving their spine to the right to maintain their center of mass over the seat to maintain their balance. Figure 13 shows a marked increase in the right femur pitch corresponding to the lifting of the right buttock while the decrease in the left femur pitch reflects more weight being shifted to the left buttock. Figure 14 shows that in a left lean, CoP_x_ moves from the center of the chair in the negative direction (towards the left). CoP_y_ begins behind the center of the seat pan and again moves in the positive direction (forward), but not as much as in the forward lean. The CoP vector’s magnitude smoothly increases, and its final angle of approximately 165° reflects that lean was both to the left and forward. Figure 15 shows that an increase in the sternum pitch corresponds to an increase in CoP_y_, whereas an increase in sternum roll corresponds to a decrease in CoP_x_. Figure 16 shows that as the compound angle of lean and CoP vector’s magnitude increase, the proportional pressure under the right IT decreases, whereas the proportional pressure under the left IT increases.

Results for a rightward lean were generally symmetric to those for the leftward lean and are therefore included in the [42].

The equivalent graphs for all participants under each test condition can be found in the [42]. Although slightly noisier and reflecting less extensive leans in each direction, the graphs for the MWUs are very similar to those for the AB participants.

### 3.2. Fitted Linear Equations of SIP–CoP Relationship

**Figure 17:**
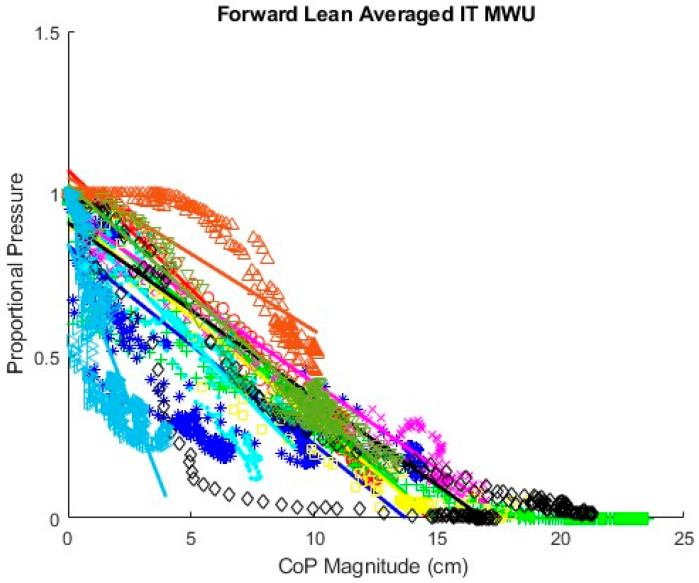
Fitted lines for MWU participants’ forward lean.

**Figure 18:**
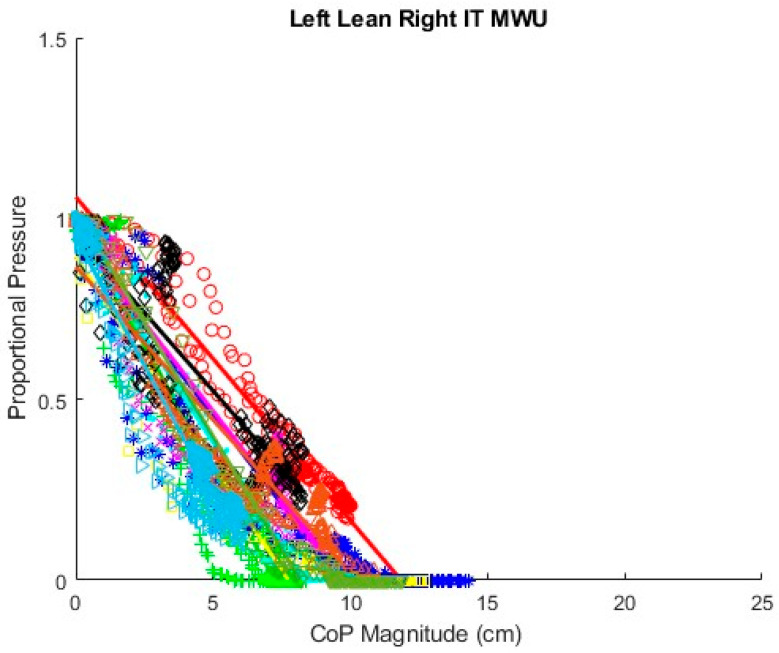
Fitted lines for MWU participants’ left lean.

Figure 17 displays proportional SIP and CoP magnitude data collected for the repeated forward leans of all 10 MWUs with a regression line fit for each participant. Line colors correspond with data point marker colors for each participant. The slopes of the regression lines appear substantially different between participants, indicating that the required change in CoP to achieve a given reduction in proportional SIP varies between participants. Figure 18 similarly shows proportional SIP and CoP magnitude data for the leftward leans. The regression lines again show differing slopes between participants. Graphs of the data with regression lines for the other experimental conditions can be found in the [42]. All show substantial differences in the slope of the SIP–CoP relationship between participants. Appendix A contains the slope, intercept, and coefficient of determination (R^2^) for each regression line, the lowest of which was 0.85, indicating reasonable fit.

**Figure 19:**
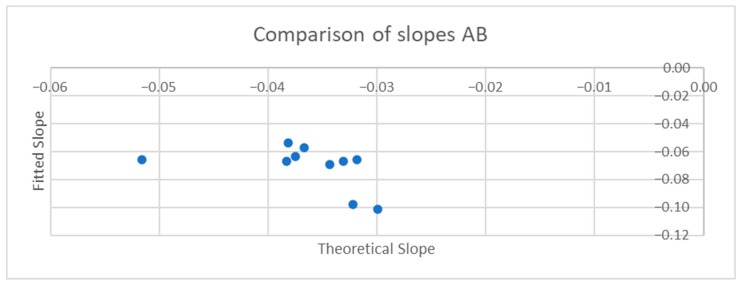
Comparison of theoretical and fitted linear coefficients for AB participants’ forward lean.

**Figure 20:**
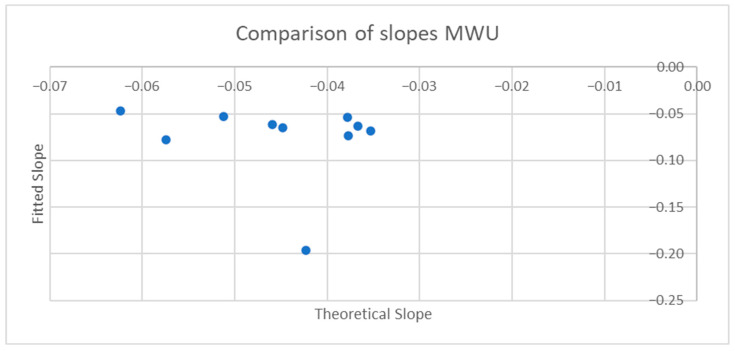
Comparison of theoretical and fitted linear coefficients for MWU participants’ forward lean.

**Figure 21:**
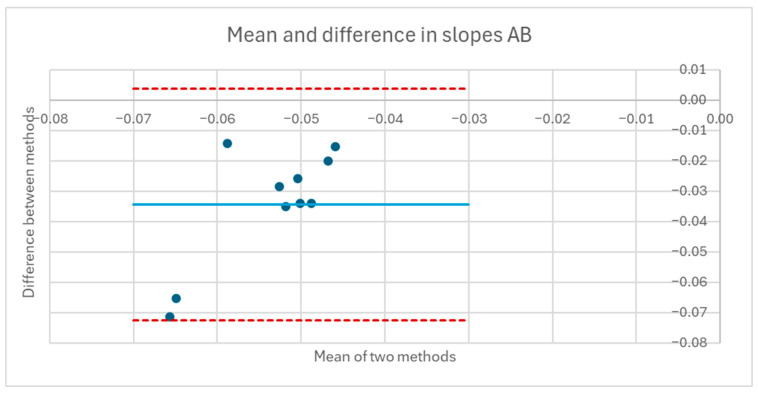
Mean and difference in theoretical and fitted linear coefficients for AB participants’ forward lean.

**Figure 22:**
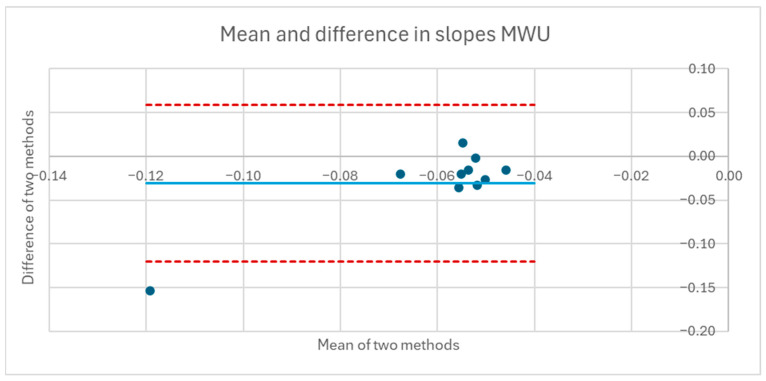
Mean and difference in theoretical and fitted linear coefficients for MWU participants’ forward lean.

Table 1 and Table 2 compare the slopes of the forward leans for each AB and MWU participant obtained from the regression lines against the theoretical slopes calculated from each participant’s body mass distribution and anatomical measurements. The experimentally determined slopes were generally larger in magnitude, corresponding to a greater reduction in proportional SIP for a given change in CoP magnitude than would be expected from the theoretically determined slope. Figure 19 and Figure 20 plot the experimentally determined slope for each participant against the theoretically derived slope with little pattern being evident. Figure 21 and Figure 22 visualize the mean and difference between experimentally and theoretically determined slopes for AB and MWU participants, respectively. The negative value of the mean difference indicates the steeper downward slope, on average, of the experimentally determined values.

### 3.3. Linear Mixed-Effects Model

Table 3 gives a comparison of the AIC and BIC for several linear mixed-effects models of data from the AB participants. AIC and BIC being lowest for the model including CoP, PR, and their interaction in the random effects structure suggests that this model best represents the underlying processes. Including more factors in the random effects structure resulted in overfitting.

Table 4 presents the results of the model for AB participants with the lowest AIC and BIC—i.e., the one including CoP, PR, and their interaction in the random effects structure. The estimate of −0.0685 for CoP indicates that for the base condition of forward lean, averaged across all participants, the proportional SIP will decrease by 0.0685 for each 1 cm increase in CoP magnitude. The effect of other factors on the relationship between proportional SIP and CoP magnitude are given by the interaction terms. That is, for a leftward leaning PR, for each 1 cm increase in CoP magnitude, the proportional SIP will decrease by an additional 0.0307 for a total reduction of 0.0992 per centimeter increase in CoP magnitude. For a rightward-leaning PR, the additional change in proportional SIP is 0.0297, for a total of 0.0982 per centimeter.

The estimate of −0.0100 for the interaction of CoP by “sit up” starting position indicates a systematic difference between these measurement conditions. Specifically, the proportional SIP decreases by 0.0685 per centimeter for the trials starting at the backrest and by 0.0785 per centimeter for the trial starting in the upright-seated position.

The estimate of 0.0008 for the CoP by cushion interaction, compared with the estimate of 0.0685 for CoP suggests that the type of cushion has only a very small influence on the CoP–SIP relationship. Table 5, which gives the effects estimates for the maximal random effects structure and therefore the most conservative *p* values, gives the *p* value for the CoP by cushion interaction as 0.857, suggesting that it is insignificant.

Table 6 compares the AIC and BIC of several models of MWU data with different random effects structures. As with the AB participants, the lowest AIC and BIC occur with the model including CoP, PR, and their interaction in the random effects structure.

The model using only data from the MWU participants (Table 7) gives an estimate of −0.0758 change in proportional SIP for each 1 cm increase in CoP magnitude, which is well within the confidence limits of the estimate for the AB participants. The estimates of −0.0333 and −0.0615 for the additional change in slope when executing, respectively, leftward- and rightward-leaning PR, are directionally similar to the results for AB participants but show a somewhat greater effect for the rightward lean.

Table 8 gives the AIC and BIC for several models constructed with data from both AB and MWU participants. The model that does not include participant type as a factor has the lowest AIC and BIC, suggesting this factor is not significant. In Table 9, the estimate for the CoP by type interaction is −0.0065, compared to −0.0697 for the estimate of CoP, and the *p* value for the interaction is 0.405, also suggesting the factor is not significant. It is therefore appropriate to use the model in Table 10 that does not include participant type. This model, logically, gives estimates for CoP and its interactions with leftward and rightward PR that are between those in the AB-only and MWU-only models.

### 3.4. Relationship of CoP and Trunk Angle

**Figure 23:**
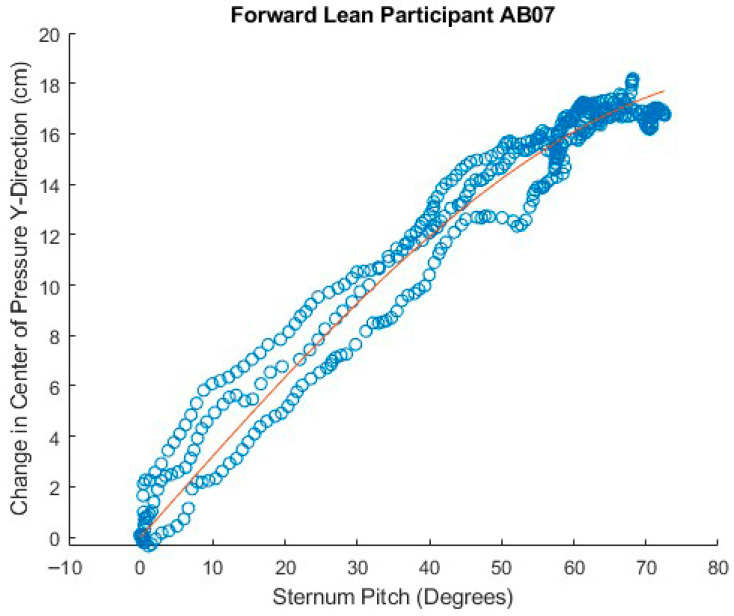
Good fit of forward lean data to angle model equation.

**Figure 24:**
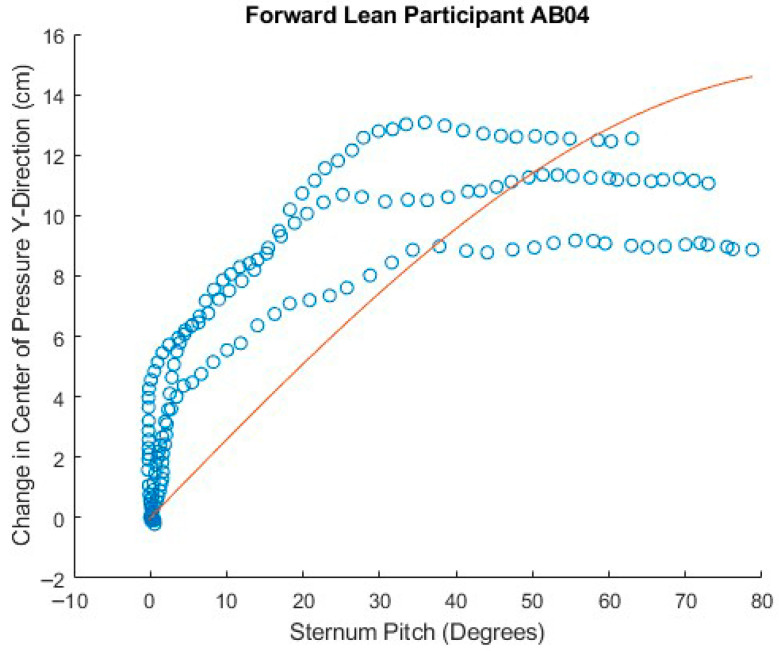
Poor fit of forward lean data to angle model equation.

**Figure 25:**
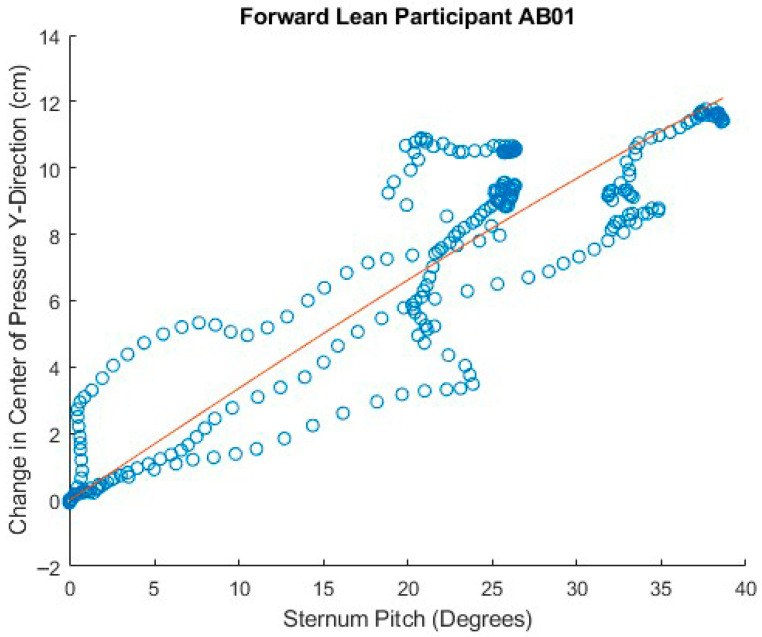
Inconsistent forward lean results in poor fit to angle model equation.

Figure 23, Figure 24 and Figure 25 show attempts to fit the sine function to the data for sternum pitch and change in CoP in the y-dimension while the participant executes a forward lean. In Figure 23, the function fits the data relatively well. In Figure 24, the CoP changes faster than would be expected from the simple lean model. If the participant were to have supported themselves during the lean by placing their hands on their knees, their weight would shift forward, bringing the CoP forward ahead of the sternum pitch. As the participant completed the forward lean, the sternum pitch would continue to increase while having less effect on the CoP. Figure 25 shows three significantly different behaviors as the participant executes the lean, some of which are difficult to interpret.

**Figure 26:**
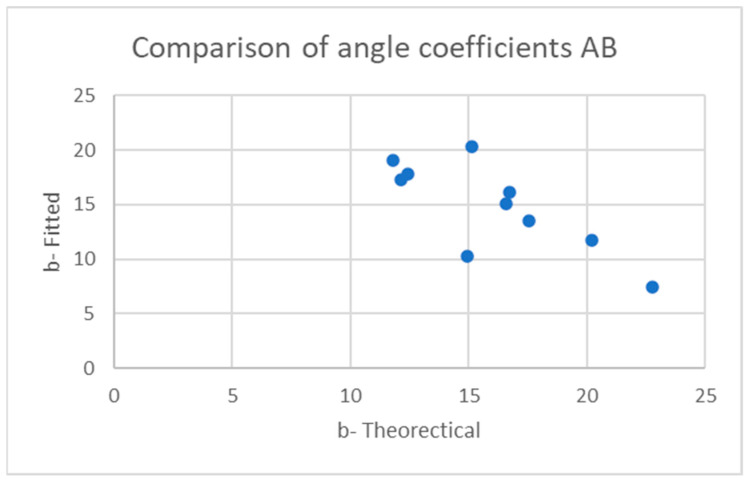
Comparison of theoretical and fitted b coefficients for AB participants’ forward lean.

**Figure 27:**
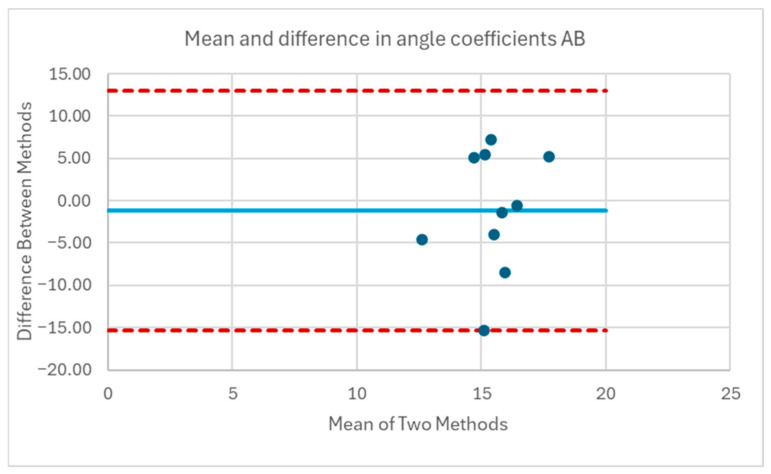
Mean and difference in theoretical and fitted b coefficients for AB participants’ forward lean.

**Figure 28:**
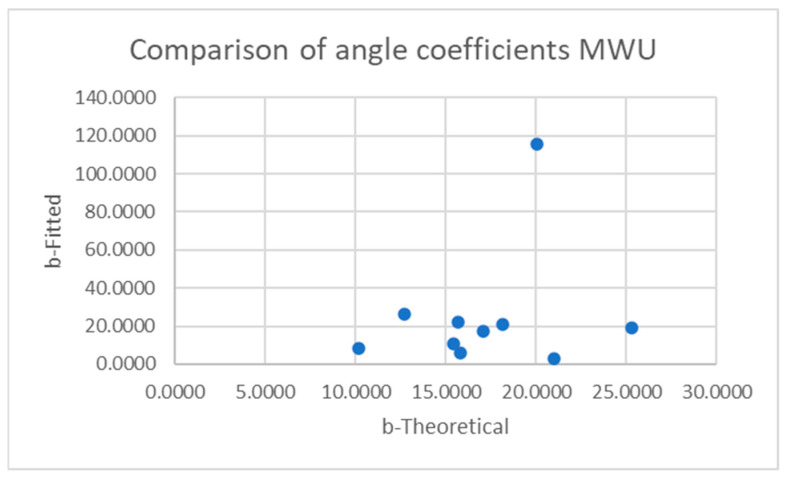
Comparison of theoretical and fitted b coefficients for MWU participants’ forward lean.

**Figure 29:**
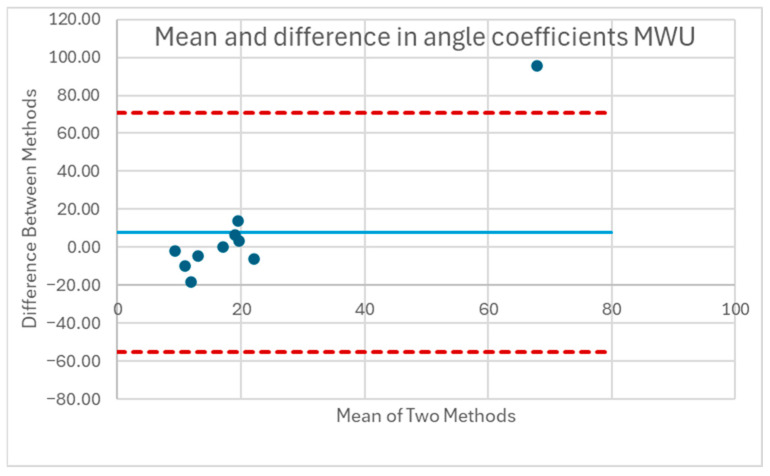
Mean and difference in theoretical and fitted b coefficients for MWU participants’ forward lean.

Table 11 and Table 12 give the best fit coefficients for the sine function relating change in CoP to sternum pitch during a forward lean as well as the expected values calculated from each participant’s mass distribution. For Table 11, the four test conditions for the AB participants were combined. Individual fitted coefficients for each test condition—e.g., foam cushion–backrest starting position—can be found in Appendix A. The R^2^ values indicate that in many cases, the equation was a poor fit to the data. Figure 26, Figure 27, Figure 28 and Figure 29 compare the fitted coefficient to the value expected from the theoretical model for AB and MWU participants, showing no discernible pattern for the deviation between the two.

## 4. Discussion

Across participants, proportional SIP can be seen to decrease with increasing CoP magnitude for both ITs during forward-leaning PR. For a leftward-leaning PR, pressure decreases sharply under the right IT and rises moderately under the left, while the opposite is true for a rightward-leaning PR. These findings correspond with expectations as during a sideways lean, weight is transferred from one side to the other along with being shifted forward; the forward shift is the reason that there is only a moderate increase in SIP on the leaning side rather than increasing as much as the opposite side decreases.

The coefficients of determination, R^2^, for the fitted regression lines, with a minimum of 0.850 and the majority being considerably higher, indicate that the relationship between proportional SIP and CoP magnitude can be reasonably represented by a linear equation for forward leans as well as for leftward and rightward leans, at least over the first 95% of the SIP reduction. Graphical representation of the relationship for each experimental condition, e.g., foam cushion–sitting up initial position–forward lean, suggests that the slope of the relationship varies by participant. Comparing linear mixed-effects models, the AIC and BIC are lowest for the models that include CoP, PR, and their interaction in the random effects structure, also indicating that the slope of the SIP–CoP relationship varies by participant for both forward and sideways leans. In part, this variation in slope is likely due to the variation in body segment lengths and mass distributions among the participants as predicted by the mathematical model. Additionally, the slope may vary due to differences in the ways the participants executed the leans. For example, if the participants supported themselves with their hands on their knees or on some part of the wheelchair, the extent to which weight is transferred off the seat would affect the SIP independent of changes in CoP. For the leftward and rightward leans, the precise direction of the lean, i.e., the ratio between the forward-leaning component and sideways-leaning component, will affect the relationship along with the extent to which the IT is lifted as opposed to the sideways shift in weight coming from a curvature of the spine. Beyond providing some instruction on how to execute the three types of PR, the ways in which participants executed each PR were not controlled in order to best capture the variety of PR techniques that might be practiced by MWUs, so it is not surprising to find some variation between participants. The wider confidence limits in the MWU model indicate greater variation in the slope of the SIP–CoP relationship among the MWUs than among the AB participants, which may be due either to greater variation in body mass distribution or in PR technique compared to the AB participants.

The linear models for AB and MWU participants, calculated independently, show a slightly (11%) greater magnitude for the estimate of CoP for MWU compared to AB participants, but both estimates were well within each other’s confidence limits and therefore not statistically different. In both models, the estimates for the interactions of CoP by PR Left and CoP by PR Right were at least 43% of the magnitude of the CoP estimate, which makes these effects practically significant in addition to their *p* values indicating statistical significance. For both groups, leaning to either side required less change in CoP magnitude than did leaning forward to achieve the same reduction in proportional SIP. The interaction effect of a leftward lean for the MWU participants (−0.0333) is very similar to that for both leftward (−0.0307) and rightward (−0.0297) leaning PR for AB participants, while that for a rightward PR (−0.0615) is notably larger. The confidence interval for the MWU rightward PR is also wider. The greater variation may be due to an asymmetrical lack of trunk control for some MWU participants.

The interaction of CoP by cushion type suggests that the cushion has very little influence over the relationship between SIP and CoP. Although different cushion materials and construction distribute the user’s weight differently, some cushions may do so in a proportional way. That is, for these cushions, a reduction by 50% in force should result in a reduction by 50% in SIP. Stated differently, considering proportional SIP, as we do here, rather than absolute reduction in SIP, the effects of the cushion are canceled. What differences exist may result from how well the pressure mat conforms to each cushion during the lean as the cushion is deformed. Contrarily, Sonenblum et al. [35] found that cushion type had an effect on pressure reduction for various PR. However, that study used a pressure sensor attached to the skin rather than a pressure mat. It also found the largest difference at the extreme lean angles, where the cushion response may no longer be proportional. Fitting a first-order linear response, while supported by both the theoretically derived model and SolidWorks simulation, precludes identifying any non-proportional responses.

There are several nonexclusive reasons why the relationship may not be linear over the entire extent of the lean. As previously noted, the transfer of weight through the arms and hands either to the knees or to some part of the wheelchair’s frame will affect both the location of CoP and SIP. The model assumed that the distal ends of the upper legs, at the knees, were free to move. To a large extent, this is correct, as leaning the torso forward causes the upper legs’ distal ends to sink further into the cushion. This behavior is reflected by the change in pitch measured on both femurs. However, as the knees move downward, more force will be transferred through the lower legs to the footplates until the knees can no longer move. At that point, the reduction in SIP for a given increase in CoP magnitude will be reduced. Adding instrumentation to the footplates could help characterize this behavior. The model assumed a cushion with uniform elasticity; if such is not the case, nonlinearities in the response are most likely to be evident at extreme lean angles. A more detailed, anthropometrically correct model of the body–cushion system may provide a basis for alternative curves to be fit to the data, although no single model would account for the variations in PR execution between participants.

The estimate of the CoP with starting condition interaction being a significant percentage of the CoP estimate was unexpected. It is possible that using the maximum SIP to normalize the responses between trials may not have sufficiently aligned their responses. Alternatively, it may be that in the trials beginning at the backrest, participants were already transferring some weight to the chair frame or the footrests via the knees and lower legs by the time they reached the point of maximum SIP; whereas in the upright-sitting position, a higher proportion of their weight was on the seat, and any transfer of weight off of the seat happened during the lean. It is therefore important in comparing the relationship between SIP and CoP to account for the user’s starting position and posture.

The models of the combined AB and MWU data suggest that it is reasonable to group the participant types together and omit type as a factor. According to that model, the proportional SIP changes by −0.0730 per centimeter increase in CoP for a forward lean, −0.1040 per centimeter for a leftward lean, and −0.1208 per centimeter for a rightward lean.

The slope estimates for the SIP–CoP relationship can be used to calculate threshold values of CoP magnitude for whatever proportional decrease in pressure is deemed an effective PR by simply dividing the proportional change by the slope. For example, achieving a 90% reduction in pressure would require a CoP magnitude of 12.33 cm for a forward lean, 8.65 cm for a leftward lean, and 7.45 cm for a rightward lean. As proposed in this team’s previous work on classification of PR using changes in CoP [34], the angle of the CoP vector can be used to determine which threshold to apply. These slope values are, however, the average responses across all participants. As the current study shows that the slope of the SIP–CoP relationship varies by individual, calibrating threshold values for each individual would result in more accurate determination of PR effectiveness. Individual calibration is also necessary to account for the fact that not all wheelchair users can lean far enough to achieve 80% or 90% pressure reduction. Calibration values and a linear relationship can be used to interpolate SIP reduction for smaller changes in CoP, but cannot be used to extrapolate larger reductions than measured for an individual. In most setups, there would be an additional offset in the CoP magnitude vector to account for travel from the backrest to upright-sitting.

The data for sternum pitch and change in CoP generally showed a poorer fit to the predicted equation than did the data for SIP and CoP magnitude. The simple model assumes a rigid trunk segment connected to a stationary upper legs segment with just a hinged joint. If there is some curvature to the spine as the participant leans forward, considering only the sternum pitch will overestimate the change in CoP. However, a model that included a linear combination of sine functions for the sternum and pelvis pitches (not presented) did not provide a better fit to the data. Supporting any amount of the torso weight through hands resting on the knees or wheelchair frame would also change the relationship between the sternum pitch and the change in CoP. These observations suggest that estimates of trunk angle—whether measured by body-worn sensors, motion-tracking cameras, or clinician observation—may not provide good estimates of SIP reduction. During some of the tests, it was noted, and observation of the collected data corroborates, that the XSENS sensors may have moved during the leans, making the angle data less reliable. In addition, in several trials, it appears that the angle data was not properly time-aligned despite the trigger. Given this poor fit for the simple forward lean model and that the angle relationship is not directly applicable to the development of the real-time pressure relief algorithm for the load cell-based system, no further model fitting was attempted with the XSENS data.

### Limitations

The study only included 20 participants, only 10 of whom were MWUs, which might not provide an accurate estimate of the SIP–CoP relationship for the entire population. Furthermore, all of the participants who were MWUs were active individuals with little or no upper limb impairment. There are MWUs still capable of performing independent weight shifts who have less trunk control and less upper limb function than our participants. Such individuals’ methods for accomplishing PR may alter the slope of the SIP–CoP relationship. Similarly, individuals with pelvic obliquity, scoliosis, or other conditions may produce different results. However, since the study findings suggest that a PR algorithm would achieve better results if calibrated to each participant, finding a population mean is less of a concern for the proposed application.

All of the participants used the same study wheelchair with the only adjustments made being blocks added to the footrests. This meant that the setup was not optimal for all of the participants, which may have affected the way in which they performed their PRs. Calibration of the system to the individual with their own set up should mitigate this concern for clinical applications. Using only one wheelchair set up also means that the slopes estimated in this study may not apply to other setups. As with the small sample size, the wide range of slopes across participants and difference between slopes with the same participant starting at the backrest or sitting upright already indicate that the system should be calibrated to each user in their own setup.

Only two cushions were tested systematically; therefore, the finding that SIP–CoP relationship did not vary by cushion may not be valid for all cushion types. Furthermore, fitting the data to a first-order equation necessarily neglected any nonlinear response of the cushions. Including the total weight recorded by the load cells, i.e., the total weight on the seat, would be a more complicated model but may better fit the data and account for some of the variation in PR techniques between participants. Similarly, reducing the dimensionality of the model by considering the single value of CoP magnitude rather than separate changes in CoP_x_ and CoP_y_ meant the model could not account for the effect of precise lean direction on proportional SIP.

Using the bed-sized pressure mat may not have accurately measured the body mass distribution of the participants. While using a compliant surface under the mat and allowing settling time helped to even out the response, the body may not lie perfectly flat, distorting the pressure distribution. The most severe cases were noted among the MWUs, some of whom had contractures at the knees such that they could not straighten their legs. If the knee is raised above the pressure mat, the weight of the leg is transferred toward the hip and the heal, which results in the upper leg CoM being measured too close to the hip and a likely underestimation of the upper leg mass. Although incorrect measures of mass distribution do not influence the estimates of the SIP–CoP or CoP–sternum angle relationships derived from the participant lean data, they may prevent the estimates from matching the values calculated from the mathematical model.

## 5. Conclusions

Across participants and PR types, the relationship between proportional SIP and CoP was found to be reasonably well represented by a first-order linear equation. The slope of this relationship was steeper for leftward and rightward leans than for forward leans, indicating that there is a greater reduction in SIP for a given change in CoP when performing a sideways lean as compared to a forward lean. Within PR type, the slope was found to vary by participant, suggesting that the best estimates of proportional SIP would be obtained by calibrating the system for each user. These user-specific slopes could then be used to set thresholds for what a real-time pressure relief algorithm would count as an effective PR based on reduction in SIP. The system could be used in a longitudinal study to determine what degree and frequency of PR is truly effective in preventing PI. Since the responses are proportional, the measurements of smaller changes in CoP could also be used to monitor the constant movements that some MWUs execute in order to study the concept of active sitting.

## Figures and Tables

**Table 1:** Comparison of theoretical and fitted linear coefficients for AB participants’ forward lean.

Participant	Theoretical Slope	Slope	Intercept	R^2^
AB01	−0.05162	−0.06591	0.98773	0.93977
AB02	−0.03219	−0.09759	0.97311	0.95999
AB03	−0.02995	−0.10138	1.05913	0.94237
AB04	−0.03435	−0.0693	0.93791	0.94844
AB05	−0.03749	−0.06334	1.01136	0.96431
AB06	−0.03181	−0.06572	0.9441	0.89138
AB07	−0.03817	−0.05364	0.9844	0.87014
AB08	−0.03831	−0.06694	0.91714	0.90519
AB09	−0.0331	−0.06706	0.99145	0.95141
AB10	−0.03672	−0.05684	0.89341	0.92868

**Table 2:** Comparison of theoretical and fitted linear coefficients for MWU participants’ forward lean.

Participant	Theoretical Slope	Slope	Intercept	R^2^
MWU1	−0.03775	−0.07337	1.07167	0.99536
MWU2	−0.04481	−0.0654	0.97095	0.96031
MWU3	−0.04591	−0.06132	0.83986	0.87788
MWU4	−0.05747	−0.07792	0.9343	0.96228
MWU5	−0.05121	−0.05298	0.94567	0.98696
MWU6	−0.03667	−0.06352	0.93615	0.96428
MWU7	−0.03778	−0.05397	0.90967	0.89173
MWU8	−0.06232	−0.04737	1.05025	0.95165
MWU9	−0.03529	−0.06839	1.02774	0.98899
MWU10	−0.04229	−0.19622	0.84576	0.92245

**Table 3:** Comparison of linear models for AB participant data.

Model	AIC	BIC
Model 1: (1)	−47,345.73	−47,243.34
Model 2: (1 + CoP)	−48,409.07	−48,289.61
Model 3: (1 + CoP + PR + CoP:PR)	−55,301.49	−55,028.45
Model 4: maximal CoP interactions	−50,469.61	−50,196.57

**Table 4:** Linear model of AB participant data (1 + CoP + PR + CoP:PR).

Effect	Estimate	Std. Error	df	t Value	Pr (>|t|)	CI_Low	CI_High
(Intercept)	0.5885	0.0241	10.0271	24.3704	2.95 × 10^−10^	0.5411	0.6358
CoP	−0.0685	0.0040	9.9622	−17.1430	1.01 × 10^−8^	−0.0763	−0.0606
Cushionfoam	0.0024	0.0012	33,931.7266	1.9643	4.95 × 10^−2^	0.0000	0.0048
Positionsitup	−0.0881	0.0012	37,048.4089	−71.7617	0.00 × 10^0^	−0.0905	−0.0857
PRleft	−0.1614	0.0358	9.9913	−4.5049	1.14 × 10^−3^	−0.2317	−0.0912
PRright	−0.1421	0.0346	9.9978	−4.0999	2.15 × 10^−3^	−0.2100	−0.0741
CoP*Cushionfoam	0.0008	0.0003	32,564.4731	2.4160	1.57 × 10^−2^	0.0001	0.0014
CoP*Positionsitup	−0.0100	0.0003	36,639.4145	−30.2073	5.14 × 10^−198^	−0.0107	−0.0094
CoP*PRleft	−0.0307	0.0072	9.9491	−4.2419	1.73 × 10^−3^	−0.0448	−0.0165
CoP*PRright	−0.0297	0.0071	9.9656	−4.1741	1.92 × 10^−3^	−0.0437	−0.0158

**Table 5:** Linear model of AB participant data maximal CoP interactions.

Effect	Estimate	Std. Error	df	t Value	Pr (>|t|)	CI_Low	CI_High
(Intercept)	0.5937	0.0184	10.1038	32.2004	1.62 × 10^−11^	0.5576	0.6299
CoP	−0.0654	0.0043	9.9902	−15.2762	2.97 × 10^−8^	−0.0737	−0.0570
Cushionfoam	0.0076	0.0014	36,569.6559	5.6178	1.95 × 10^−8^	0.0049	0.0102
Positionsitup	−0.0893	0.0013	33,586.1300	−66.5885	0.00 × 10^0^	−0.0919	−0.0867
PRleft	−0.1509	0.0017	31,941.1661	−87.5674	0.00 × 10^0^	−0.1543	−0.1475
PRright	−0.1549	0.0017	30,417.8796	−92.1185	0.00 × 10^0^	−0.1582	−0.1516
CoP*Cushionfoam	0.0006	0.0033	10.0396	0.1853	8.57 × 10^−1^	−0.0058	0.0070
CoP*Positionsitup	−0.0127	0.0022	10.2020	−5.8788	1.44 × 10^−4^	−0.0169	−0.0084
CoP*PRleft	−0.0272	0.0033	9.8783	−8.3229	8.99 × 10^−6^	−0.0336	−0.0208
CoP*PRright	−0.0302	0.0045	9.9884	−6.6897	5.46 × 10^−5^	−0.0391	−0.0214

**Table 6:** Comparison of linear models for MWU participant data.

Model	AIC	BIC
Model 1: (1)	−7085.772	−7031.53
Model 2: (1 + CoP)	−7434.973	−7367.17
Model 3: (1 + CoP + PR + CoP:PR)	−10,452.386	−10,262.54

**Table 7:** Linear model of MWU participant data (1+ CoP + PR + CoP:PR).

Effect	Estimate	Std. Error	df	t Value	Pr (>|t|)	CI_Low	CI_High
(Intercept)	0.6114	0.0717	9.9459	8.5215	6.99 × 10^−6^	0.4708	0.7520
CoP	−0.0758	0.0128	9.7606	−5.9101	1.64 × 10^−4^	−0.1009	−0.0507
PRleft	−0.1414	0.0591	9.9512	−2.3940	3.78 × 10^−2^	−0.2572	−0.0256
PRright	−0.2555	0.0775	9.9173	−3.2983	8.13 × 10^−3^	−0.4074	−0.1037
CoP*PRleft	−0.0333	0.0101	9.6775	−3.2856	8.57 × 10^−3^	−0.0531	−0.0134
CoP*PRright	−0.0615	0.0167	9.8548	−3.6900	4.28 × 10^−3^	−0.0942	−0.0288

**Table 8:** Comparison of linear models for all participant data.

Model	AIC	BIC
Model 1: (1)	−28,633.77	−28,552.29
Model 2: (1 + CoP)	−29,428.31	−29,330.53
Model 3: (1 + CoP + PR + CoP:PR)	−36,353.55	−36,109.10
Model 4: without Type; maximal random slopes	−36,353.97	−36,125.83

**Table 9:** Linear model of all participant data including participant type.

Effect	Estimate	Std. Error	df	t Value	Pr (>|t|)	CI_Low	CI_High
(Intercept)	0.5917	0.0544	26.1189	10.8791	3.38 × 10^−11^	0.4851	0.6983
CoP	−0.0697	0.0079	25.4845	−8.7997	3.36 × 10^−9^	−0.0853	−0.0542
PR left	−0.1807	0.0409	19.6090	−4.4145	2.78 × 10^−4^	−0.2609	−0.1004
PR Right	−0.2462	0.0579	19.5487	−4.2555	4.04 × 10^−4^	−0.3596	−0.1328
Type MWU	−0.0930	0.0510	20.0120	−1.8220	8.34 × 10^−2^	−0.1931	0.0070
CoP*PRleft	−0.0310	0.0070	19.5737	−4.4363	2.66 × 10^−4^	−0.0447	−0.0173
CoP*PRright	−0.0478	0.0099	19.4957	−4.8328	1.08 × 10^−4^	−0.0672	−0.0284
CoP*TypeMWU	−0.0065	0.0076	20.0459	−0.8509	4.05 × 10^−1^	−0.0213	0.0084

**Table 10:** Linear model of all participant data without participant type.

Effect	Estimate	Std. Error	df	t Value	Pr (>|t|)	CI_Low	CI_High
(Intercept)	0.5452	0.0492	19.6238	11.0853	6.77 × 10^−10^	0.4488	0.6416
CoP	−0.0730	0.0070	19.1032	−10.4345	2.50 × 10^−9^	−0.0867	−0.0593
PRleft	−0.1804	0.0410	19.6157	−4.4042	2.85 × 10^−4^	−0.2607	−0.1001
PRright	−0.2463	0.0579	19.5520	−4.2560	4.04 × 10^−4^	−0.3597	−0.1329
CoP*PRleft	−0.0310	0.0070	19.5776	−4.4267	2.72 × 10^−4^	−0.0447	−0.0173
CoP*PRright	−0.0478	0.0099	19.4975	−4.8331	1.08 × 10^−4^	−0.0671	−0.0284

**Table 11:** Fitted coefficients of angle–CoP relationship for AB participant data, with all forward leans combined.

All AB Trials Combined			
AB Participant Number	R^2^	b-Fitted	b-Theoretical
1	0.6890	19.0178	11.7830
2	0.5700	10.3130	14.9420
3	0.3385	7.4405	22.7535
4	0.2023	16.1360	16.7148
5	0.9140	17.2620	12.1198
6	0.7439	15.1047	16.5614
7	0.7630	17.8516	12.4357
8	0.7142	11.6912	20.2104
9	0.8212	13.4841	17.5376
10	0.7280	20.3409	15.1161

**Table 12:** Fitted coefficients of angle–CoP relationship for MWU participant data.

Participant	b-Theoretical	R^2^	b-Fitted
MWU01	15.4367	0.7130	10.8148
MWU02	15.8188	0.1244	6.0138
MWU03	10.1792	0.3185	8.4089
MWU04	12.7163	0.8715	26.3538
MWU05	15.6960	0.9375	22.3955
MWU06	25.3150	−0.2640	18.9227
MWU07	17.0734	0.8955	17.0619
MWU08	20.0420	0.6890	115.6000
MWU09	18.1155	0.7790	21.1928
MWU10	20.9811	0.3354	2.8137

## Data Availability

Data is available from the corresponding author upon reasonable request.

## Appendix A. Fitted Linear and Angle Coefficients for AB and MWU Participants

**Table A1:** Fitted linear coefficients for AB participants, air cushion, upright-sitting starting position.

Air Sit Up	Forward Lean Averaged ITs			Left Lean Right IT			Right Lean Left IT		
Participant	Slope	Intercept	R^2^	Slope	Intercept	R^2^	Slope	Intercept	R^2^
AB01	−0.091	0.971	0.987	−0.1	0.981	0.983	−0.119	0.984	0.989
AB02	−0.127	1.001	0.996	−0.105	1.012	0.986	−0.079	0.935	0.983
AB03	−0.101	1.026	0.982	−0.139	1.015	0.991	−0.105	1.023	0.987
AB04	−0.089	0.97	0.983	−0.112	1.097	0.986	−0.093	0.929	0.965
AB05	−0.082	1.025	0.989	−0.109	0.958	0.992	−0.132	0.948	0.987
AB06	−0.084	1.086	0.941	−0.106	0.963	0.975	−0.106	1.015	0.963
AB07	−0.082	0.982	0.98	−0.084	0.943	0.971	−0.092	0.954	0.975
AB08	−0.095	0.975	0.96	−0.152	1.015	0.984	−0.092	0.993	0.986
AB09	−0.092	1.053	0.99	−0.065	1.019	0.99	−0.074	0.946	0.987
AB10	−0.075	0.977	0.973	−0.147	0.937	0.974	−0.12	0.947	0.988

**Table A2:** Fitted linear coefficients for AB participants, air cushion, backrest starting position.

Air Backrest	Forward Lean Averaged ITs			Left Lean Right IT			Right Lean Left IT		
Participant	Slope	Intercept	R^2^	Slope	Intercept	R^2^	Slope	Intercept	R^2^
AB01	−0.079	1.102	0.975	−0.090	1.020	0.972	−0.084	0.954	0.991
AB02	−0.101	0.992	0.970	−0.087	0.991	0.997	−0.067	1.011	0.976
AB03	−0.125	1.027	0.993	−0.201	0.982	0.952	−0.115	0.926	0.961
AB04	−0.066	0.911	0.965	−0.092	1.088	0.981	−0.097	1.056	0.981
AB05	−0.064	1.110	0.995	−0.080	1.055	0.995	−0.101	1.113	0.979
AB06	−0.057	1.100	0.974	−0.088	1.015	0.900	−0.108	1.036	0.894
AB07	−0.066	1.269	0.971	−0.079	1.031	0.963	−0.118	1.489	0.868
AB08	−0.063	1.030	0.990	−0.134	1.014	0.966	0.032	0.802	0.850
AB09	−0.099	1.145	0.981	−0.121	1.099	0.996	−0.114	1.133	0.978
AB10	−0.055	0.938	0.969	−0.141	1.118	0.945	−0.147	1.063	0.900

**Table A3:** Fitted linear coefficients for AB participants, foam cushion, upright-sitting starting position.

Foam Sit Up	Forward Lean Averaged ITs			Left Lean Right IT			Right Lean Left IT		
Participant	Slope	Intercept	R^2^	Slope	Intercept	R^2^	Slope	Intercept	R^2^
AB01	−0.061	0.978	0.994	−0.100	1.028	0.989	−0.115	1.034	0.994
AB02	−0.092	0.988	0.994	−0.092	0.968	0.983	−0.107	0.951	0.984
AB03	−0.078	1.045	0.992	−0.101	0.963	0.976	−0.086	0.967	0.983
AB04	−0.075	0.945	0.963	−0.112	1.043	0.992	−0.117	0.990	0.983
AB05	−0.065	1.046	0.992	−0.109	1.003	0.987	−0.110	0.996	0.990
AB06	−0.086	0.983	0.983	−0.119	0.962	0.959	−0.126	1.055	0.979
AB07	−0.088	1.002	0.970	−0.082	1.010	0.995	−0.092	1.049	0.986
AB08	−0.091	0.933	0.972	−0.132	1.003	0.946	−0.112	0.951	0.946
AB09	−0.066	0.972	0.980	−0.090	1.036	0.995	−0.087	0.966	0.991
AB10	−0.093	0.963	0.985	−0.202	1.027	0.988	−0.158	0.994	0.968

**Table A4:** Fitted linear coefficients for AB participants, foam cushion, backrest starting position.

Foam Backrest	Forward Lean Averaged ITs			Left Lean Right IT			Right Lean Left IT		
Participant	Slope	Intercept	R^2^	Slope	Intercept	R^2^	Slope	Intercept	R^2^
AB01	−0.057	0.983	0.990	−0.117	1.090	0.959	−0.102	1.150	0.989
AB02	−0.084	0.981	0.992	−0.089	1.008	0.993	−0.103	1.023	0.987
AB03	−0.072	1.002	0.972	−0.093	0.869	0.936	−0.088	1.025	0.996
AB04	−0.066	1.002	0.973	−0.109	1.150	0.948	−0.120	1.031	0.945
AB05	−0.074	1.007	0.983	−0.122	0.895	0.970	−0.127	1.053	0.954
AB06	−0.085	0.950	0.990	−0.136	1.025	0.903	−0.120	1.01	0.919
AB07	−0.065	1.139	0.984	−0.093	1.191	0.975	−0.115	1.289	0.901
AB08	−0.074	1.028	0.949	−0.146	1.169	0.944	−0.127	1.064	0.902
AB09	−0.062	1.052	0.976	−0.097	1.144	0.984	−0.103	1.093	0.991
AB10	−0.058	0.991	0.990	−0.150	1.168	0.910	−0.128	1.158	0.899

**Table A5:** Fitted linear coefficients for MWU participants, backrest starting position.

MWU Backrest	Forward Lean Averaged ITs			Left Lean Right IT			Right Lean Left IT		
Participant	Slope	Intercept	R^2^	Slope	Intercept	R^2^	Slope	Intercept	R^2^
MWU1	−0.0734	1.0717	0.9954	−0.0890	1.060	0.9850	−0.1020	0.9880	0.9860
MWU2	−0.0654	0.9710	0.9603	−0.1360	0.9890	0.9610	−0.2480	1.0010	0.970
MWU3	−0.0613	0.8399	0.8779	−0.0980	0.9460	0.9340	−0.1650	0.9580	0.9560
MWU4	−0.0779	0.9343	0.9623	−0.1160	0.9640	0.9680	−0.0920	0.9180	0.9460
MWU5	−0.0530	0.9457	0.9870	−0.0980	0.9570	0.9560	−0.1420	1.0380	0.9860
MWU6	−0.0635	0.9362	0.9643	−0.1160	0.8960	0.9040	−0.1290	0.9470	0.9640
MWU7	−0.0539	0.9097	0.8917	−0.0860	0.9560	0.9330	−0.0980	0.9340	0.8840
MWU8	−0.0474	1.0503	0.95187	−0.0850	0.8670	0.9500	−0.1040	1.0510	0.9680
MWU9	−0.06840	1.0277	0.9890	−0.1250	1.0240	0.9660	−0.1250	0.9170	0.9590
MWU10	−0.1962	0.8458	0.9225	−0.1420	0.9490	0.9740	−0.1700	0.9820	0.9710

**Table A6:** Fitted coefficients of angle–CoP relationship for AB participant data.

		Foam Sit Up		Air Sit Up		Air Backrest		Foam Backrest	
Participant	b-Theoretical	R^2^	b-Fitted	R^2^	b-Fitted	R^2^	b-Fitted	R^2^	b-Fitted
AB01	11.783	0.919	20.116	0.809	16.891	0.828	16.342	0.391	23.696
AB02	14.942	0.827	16.857	0.665	8.981	0.793	8.734	0.464	14.641
AB03	22.754	0.771	8.832	0.370	14.601	0.462	6.977	0.895	5.788
AB04	16.715	0.293	17.024	0.502	28.885	0.357	11.676	0.556	24.055
AB05	12.120	0.992	14.700	0.945	20.331	0.945	18.116	0.973	17.432
AB06	16.561	0.734	21.983	0.881	13.470	0.775	19.865	0.784	12.371
AB07	12.436	0.819	13.960	0.947	18.354	0.614	21.090	0.876	19.071
AB08	20.210	0.936	11.062	0.846	10.241	0.571	23.130	0.754	12.826
AB09	17.538	0.913	13.480	0.922	9.664	0.735	16.040	0.956	15.325
AB10	15.116	0.464	16.193	0.782	19.408	0.812	22.113	0.798	22.465
